# Typhoid fever outbreak in the Democratic Republic of Congo: Case control and ecological study

**DOI:** 10.1371/journal.pntd.0006795

**Published:** 2018-10-03

**Authors:** Julii Brainard, Rob D’hondt, Engy Ali, Rafael Van den Bergh, Anja De Weggheleire, Yves Baudot, Frederic Patigny, Vincent Lambert, Rony Zachariah, Peter Maes, Donat Kuma-Kuma Kenge, Paul R. Hunter

**Affiliations:** 1 Norwich Medical School, University of East Anglia, Norwich, United Kingdom; 2 Operational Centre Brussels, Médecins Sans Frontières, Brussels, Belgium; 3 Department of Clinical Sciences, Institute of Tropical Medicine, Antwerp, Belgium; 4 Network for Application & Development of Aerospatial Remote sensing (N.A.D.A.R), Belgium; 5 Ministry of Public Health, Health District Kikwit, Bandundu, DRC; University of Otago, NEW ZEALAND

## Abstract

During 2011 a large outbreak of typhoid fever affected an estimated 1430 people in Kikwit, Democratic Republic of Congo. The outbreak started in military camps in the city but then spread to the general population. This paper reports the results of an ecological analysis and a case-control study undertaken to examine water and other possible transmission pathways. Attack rates were determined for health areas and risk ratios were estimated with respect to spatial exposures. Approximately 15 months after the outbreak, demographic, environmental and exposure data were collected for 320 cases and 640 controls residing in the worst affected areas, using a structured interview questionnaire. Unadjusted and adjusted odds ratios were estimated. Complete data were available for 956 respondents. Residents of areas with water supplied via gravity on the mains network were at much greater risk of disease acquisition (risk ratio = 6.20, 95%CI 3.39–11.35) than residents of areas not supplied by this mains network. In the case control study, typhoid was found to be associated with ever using tap water from the municipal supply (OR = 4.29, 95% CI 2.20–8.38). Visible urine or faeces in the latrine was also associated with increased risk of typhoid and having chosen a water source because it is protected was negatively associated. Knowledge that washing hands can prevent typhoid fever, and stated habit of handwashing habits before cooking or after toileting was associated with *increased* risk of disease. However, observed associations between handwashing or plate-sharing with disease risk could very likely be due to recall bias. This outbreak of typhoid fever was strongly associated with drinking water from the municipal drinking water supply, based on the descriptive and analytic epidemiology and the finding of high levels of faecal contamination of drinking water. Future outbreaks of potentially waterborne disease need an integrated response that includes epidemiology and environmental microbiology during early stages of the outbreak.

## Author summary

There was a large outbreak of typhoid fever in Kikwit, DRC, in late 2011. The outbreak started in military camps in the city but then spread to the general population. Multiple investigations were undertaken to understand how the disease spread. The worst affected areas of the city were mapped and compared to the water network. In early 2013, demographic and exposure data were collected for 320 cases and 640 controls residing in the worst affected areas, using a structured interview questionnaire to try to better understand individual risk factors. Residents of areas with water supplied via a gravity fed network were about six times more likely to have been ill with typhoid fever than residents of areas not supplied by the mains network. The most important individual risk factor was ever using tap water. Visible urine or faeces increased risk of getting typhoid but having chosen a water source because it is protected was linked to lower risk. Not handwashing and regularly sharing plates of food were also linked to less illness, but these findings may be especially subject to recall bias. The water network was also found to be heavily contaminated, including with faecal bacteria of human origin in multiple microbiological studies. Spatial, microbiological and case-control studies all implicate the water supplies in Kikwit to be unsafe and linked to spread of typhoid fever in 2011. Improvements to the mains water network in Kikwit are urgently needed to prevent future typhoid fever outbreaks.

## Introduction

Typhoid fever (TF) is an infection caused by the bacterium *Salmonella enterica* serovar typhi (*S*. Typhi). The primary symptoms are fever and related malaise, but serious complications, such as intestinal haemorrhage or perforation (1–4% of all cases [1]), encephalitis, respiratory infections and metastatic abscesses can occur. In the absence of treatment, there is a case fatality rate of 10–30%, which drops to ~1% with timely treatment [2]. Data from 2010–2013 suggested that the TF disease burden in Africa was 4.3 million cases per year (95%CI 3.7–5.1 million) [3]. Data to estimate the total case fatality rate in sub-Saharan Africa are unreliable [1,3]. The mean case-fatality rate after *S*. Typhi caused by intestinal perforation has been reported at 19.5% (95%CI 16–22%) in African countries [4].

Typhoid is a strictly human infection and spreads from one person to another especially through faecal oral or urine oral pathways including consumption of contaminated food or water. Spread is exacerbated by poor sanitation and hygiene. Outbreaks have regularly been reported, but those occurring in low income countries are not well researched. Instead, most studies have described outbreaks in industrialized settings [2]. Consequently, opportunities to reduce transmission in these low-resource settings may not be identified or properly understood.

Repeated and sometimes severe outbreaks of typhoid have occurred in the Democratic Republic of Congo [5]. After a large outbreak of typhoid in the city of Kikwit, Bandundu Province, in the year 2006, typhoid became endemic, with low but persistent numbers of cases reported annually until another large outbreak in November 2011 to early January 2012. The 2011–2012 event resulted in 1430 identified cases. Seventy-one people developed peritonitis with perforation, and 17 people died in 2011–2012. The fatality rate was 1.5% [6]. An initial descriptive epidemiological study recognised that contaminated water supplies were likely responsible for most cases in 2011 [6], but did not elucidate on transmission pathways or other risk factors for infection in the subsequent phases of the outbreak. Patients in the 2006 and 2011 outbreaks appeared to come from the same areas of the city, which led to suspicions that an infrastructure problem or spatial feature might contribute to the risk of catching TF (S1 Protocol). It was observed that 2011 attack rates were highest in military camps within the city, especially early in the outbreak. The descriptive evidence suggested that the 2011 outbreak originated in the camps and subsequently spread to the general population. Here, we provide a quantitative investigation of exposure factors linked to infection in 2011, accompanied with recommendations both for prevention and during emergency response.

## Methods

We undertook both an ecological analysis and a case-control study in order to better understand the primary transmission pathways of the epidemic and determine whether we could identify other risk factors. Based on the descriptive epidemiology and general knowledge of the epidemiology of typhoid, our primary hypotheses were 1) that the outbreak was waterborne and/or 2) that the infection spread directly from person to person.

### Study setting

**Fig 1:**
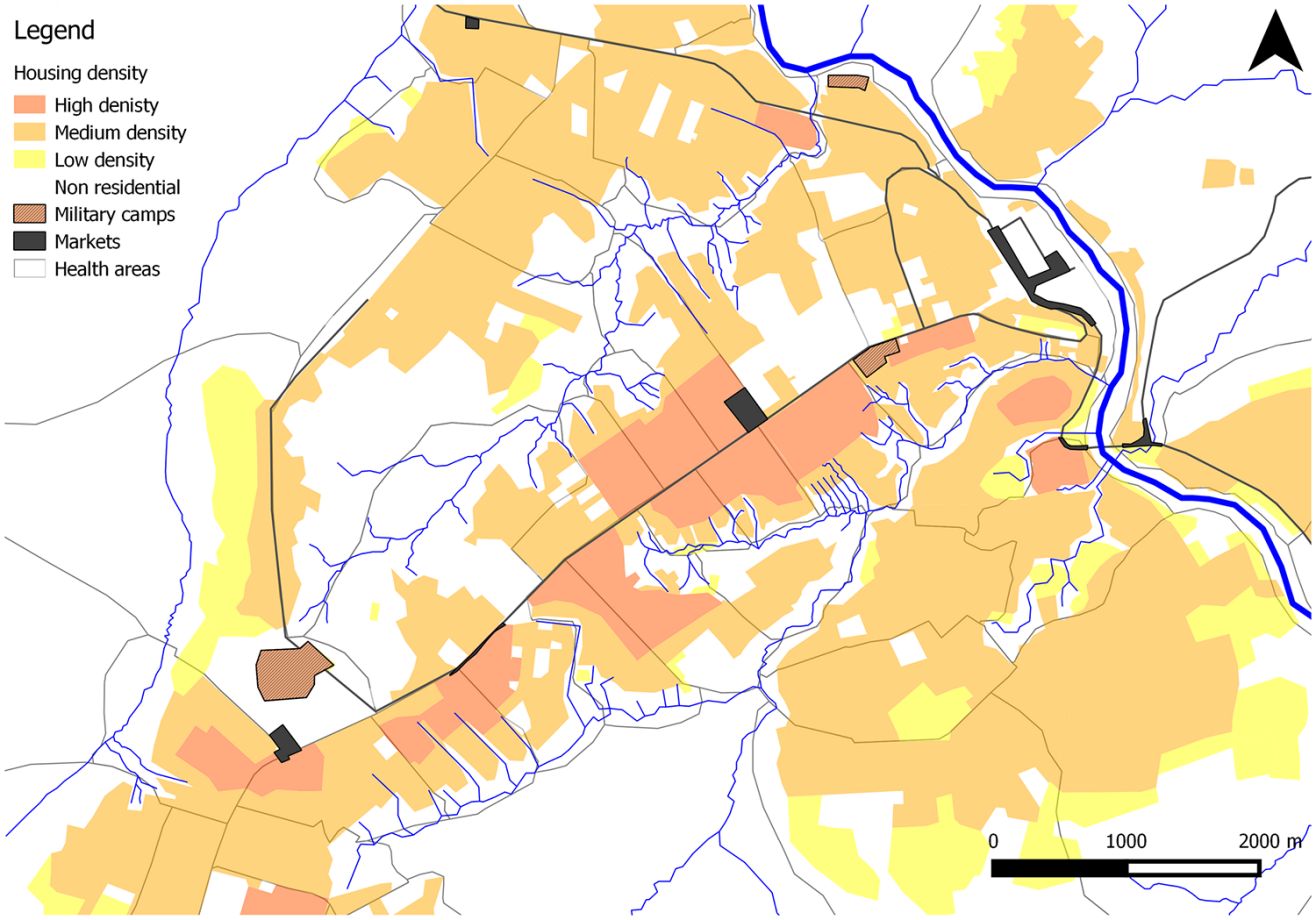
Household density map of Kikwit city, DRC, using homogenous areas visually classified as having high, medium or low housing density based on recent high-resolution satellite imagery.

Kikwit is the largest city in the Bandundu province of DRC. The estimated population in 2011 was 400,000 (Fig 1). Kikwit is located in the south-west of DRC and is an important commercial and administrative centre. In 2011, the city had one general referral hospital and was administratively divided into two health zones, north and south. Each health zone (“Zone de Santé”) was divided into health areas (“Aires de Santé”). There were 19 and 22 health areas respectively in north and south Kikwit at the time of the study. At the time of the outbreak, there were three military camps in the city, which accommodated about 2400 people: staff and families. Ngubu camp and Nsinga camp were in close proximity to the general population, while Ebeya camp was relatively separate from the city. The camp residents could be considered a highly mobile population and are mostly not of local origin. They had living conditions distinct from and mostly worse than the city’s settled residents. The military staff and families originate from many regions of DRC and speak many languages. Overall, living conditions in the three camps were poor and featured relatively high population density and poor hygiene and sanitation conditions. As a result, this study treated the three camps as three additional health areas which were analysed independently of the Aire de Santé where they were situated [6].

**Fig 2:**
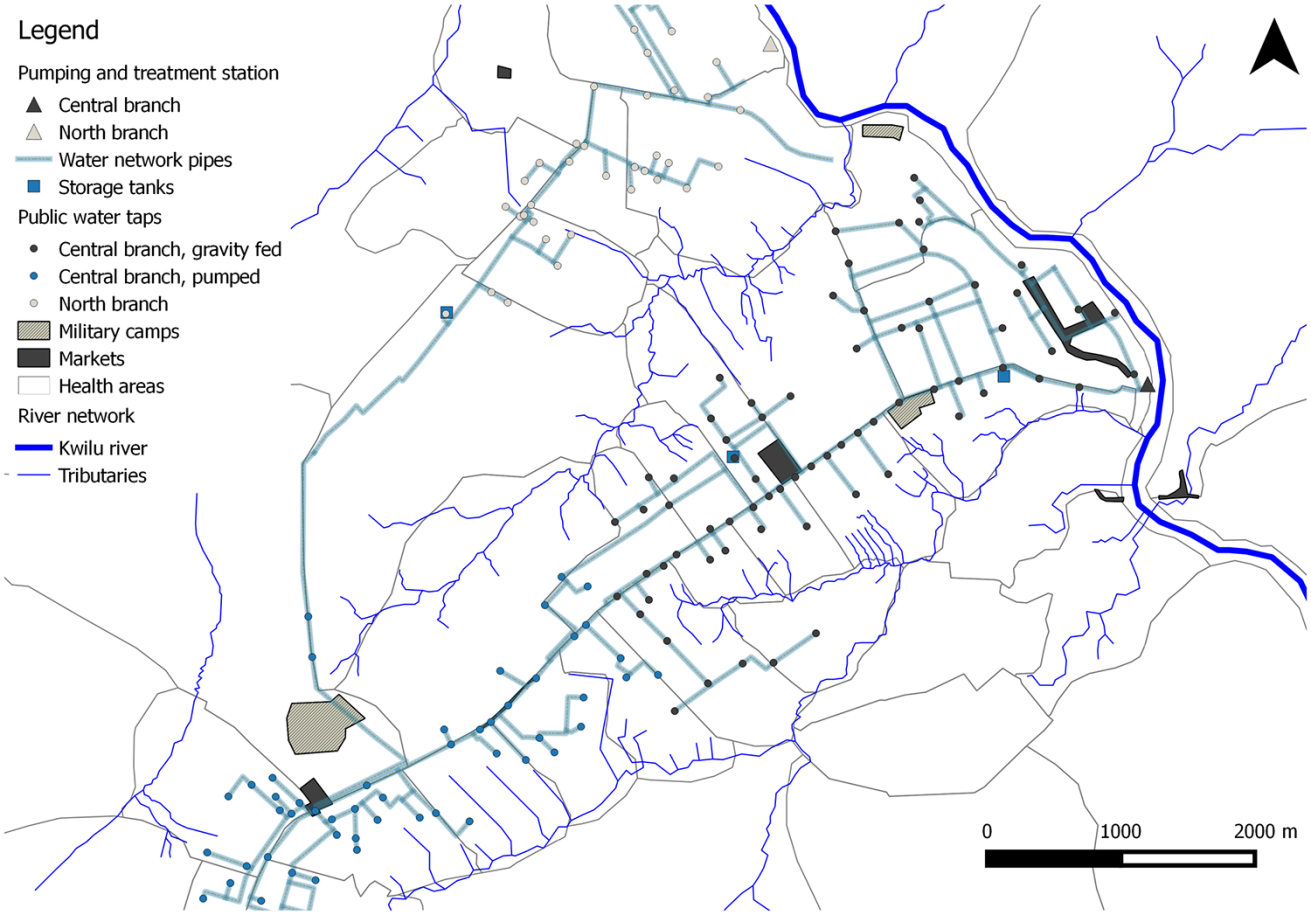
Drinking water network and sources in Kikwit, 2011–2013.

Kikwit is a hilly city with sandy soils and large erosion gullies. The city has a long wet season with an average of about 200mm of rain per month, from early September through end May. The city has centrally collected and distributed piped water, which is extracted from artesian wells, inconsistently chlorinated (S1 Report) and distributed via an aging pipe network to community tap points (standpipes, Fig 2). Water pressure is usually low throughout the network. Mains water is relatively expensive and difficult to access in parts of the city (S1 Report). Some homes have access to private wells. Surface water sources are widely available and generally free-of-charge. Surface sources are the river Kwilu, its tributaries, as well as protected and unprotected springs. No changes to the water infrastructure occurred in the time that elapsed between the 2011 outbreak and the survey dates.

Sanitation in the city is privately managed. Latrines are typically dug out by hand, and often are open air (no roof) and shared among multiple households. After a latrine fills up it is typically covered with thin soil, a mango tree is planted and a replacement latrine is dug nearby. Flooding of human waste out of active latrines is not unusual following significant rainfall events.

### Ecological analysis

Study design and protocol for the spatial analysis is in S1 Protocol. During the 2011 typhoid outbreak, The Ministry of Public Health (MOPH) created a central register line list of all cases, both suspected and confirmed. The case definitions were set by the Ministry of Public Health (MOPH); “suspected case” was any person with fever ≥ 38°C for more than three days and digestive disorders, which were defined as diarrhoea, constipation or abdominal pain, as well as a negative malaria test. A “confirmed case” was a suspected case confirmed by isolation of *S*. Typhi from blood, bone marrow or duodenal fluid. The outbreak was confirmed by the MOPH based on the results of cultures of 50 blood and stool samples, which were tested in the University Hospital in Kinshasa. From the start of the outbreak, all TF cases had their data entered into a central electronic line list developed by the MOPH. The register included patient’s name, sex, age and address. By the end of the outbreak, the line register had 1430 cases, with illness onset dates from 19 Nov 2011 to 5 January 2012. The attack rate for each of the 41 health areas and three military camps was determined using the central register line list and estimated 2011 population data. Environmental elements were mapped [7] in early-mid 2013 for each health area in Kikwit using ArcGIS software (ESRI, California, USA). Spatial attributes were assigned to an entire health area as the predominate trait for that health area. A key environmental item of interest was the predominant water distribution network that public water taps connected to in each area. The network options were: northern pump, central pump, central gravity or off network. Other spatial attributes for each health area were population density the (average) distances from each health area to the nearest camp, market, school, springs, tarred road, water point or health care clinic. Distances to these locations were of interest because these places tend to be places where people cluster and thus might pass on infection. Data on socio-economic status, sanitation statistics or water quality (such as via the WHO Joint Monitoring Programme) were unavailable at the scale of health areas. Incident risk ratios were estimated for each health area using negative binomial regression, testing for any association between single or multiple exposures to environmental attributes in the health area and attack rate. The outcome was set to be the number of cases within each health area, the model offset was the (natural log transformation of the) total population in that same area. Distances were modelled continuously under a Poisson functional form. Categories were also available that described the water source in each health area (gravity or pumped, north or central, see data in S1 Spreadsheet).

### Case-control study

Study design and protocol is item S2 Protocol. The case-control study focused on individual risk factors. The data were collected using structured household interviews between February and May 2013. Descriptive results of the data collected about cases during these interviews are described elsewhere [6]. Aspects of the structured survey as reported previously will be briefly recapped here.

**Fig 3:**
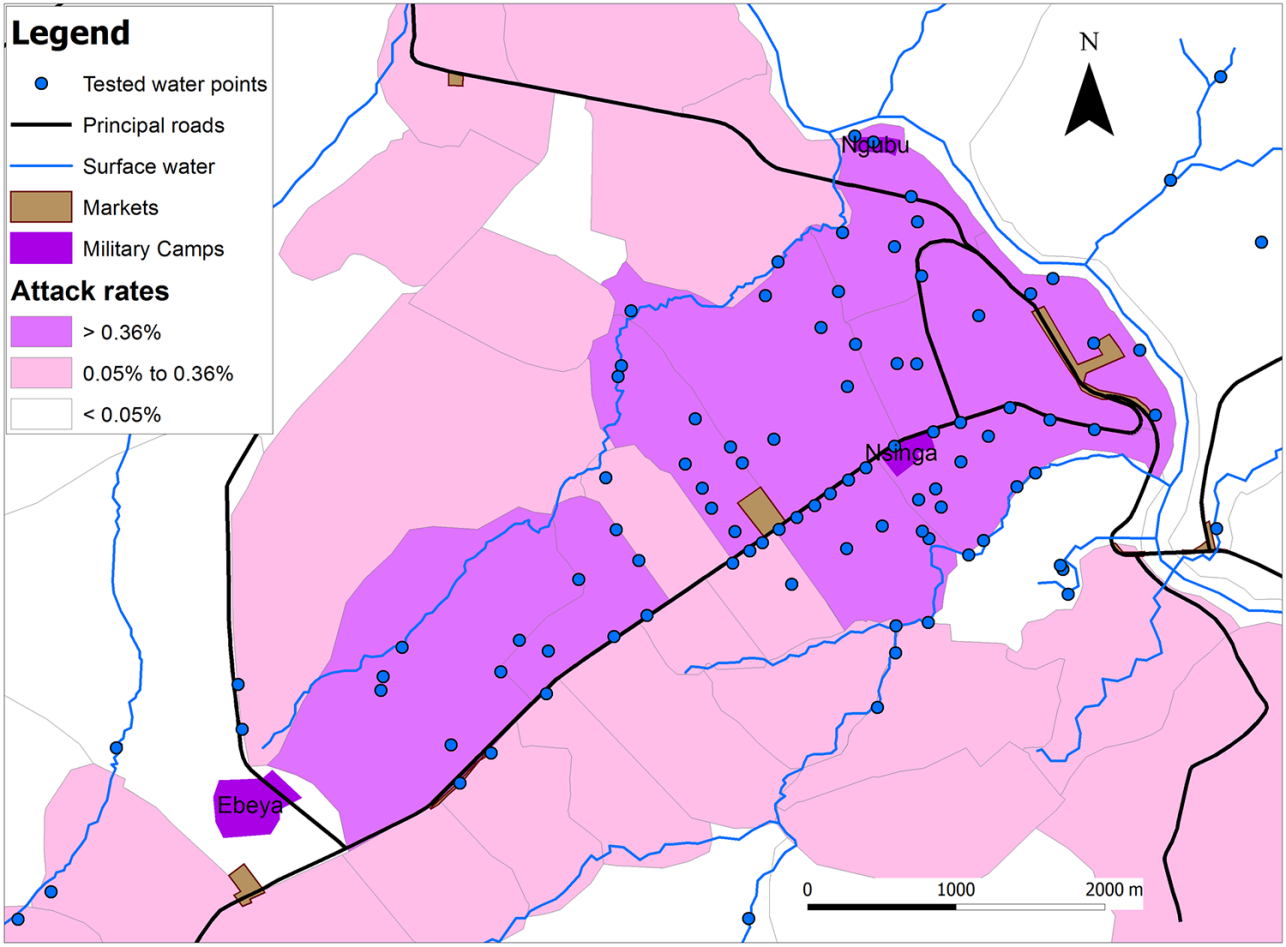
The health areas of Kikwit city, DRC, with low (<0.05%), intermediate (0.05% to 0.36%) or high (>0.36%) total attack rates during the 2011 typhoid fever outbreak as well as the city’s military camps (labelled with name), markets, principal roads and points where water was tested.

The survey was targeted at cases and controls living in the Aires de Santé with the highest attack rates during the outbreak. The case-control study was done in only these areas primarily for logistical reasons. The attack rate for each health area was estimated using the denominator from population census data. For the case control study, we surveyed residents in the eight most affected health areas (attack rates > 0.36%) while also separating out and interviewing residents of the three military camps, because the camps all had AR > 4% (Fig 3).

To identify an odds ratio of at least 1.5 with a power of 80% for risk factors present in 30% of the control population, a sample size was set at 320 cases (25% of total), frequency matched by age and sex to 640 controls.

A structured questionnaire was developed and piloted (S1 Questionnaire). Twelve interviewers and one supervisor who spoke local languages were trained to verbally administer the questionnaire. Interviewers exercised own judgement in how to translate the questionnaire from French into other languages, when required. The interviewers were trained how to record household location and its principal water source using GPS devices (Garmin GPSMAP 76). Using the recorded addresses in the line list records, typhoid cases (from the concurrent case definition) were chosen at random, using random numbers generated in MS Excel. Cases were traced and then interviewed in the community. Serology was not used to confirm recovered case status.

Two controls of the same sex, age class of 5 years interval (0–5, 6–10, 11–15, 16–20, etc.) and health area were selected per case. Selection criterion for each control was not having been suspected of typhoid during the outbreak period. After interviewing a case, interviewers decided which end of their road to treat as the starting point, and then chose two control households by rolling a die to select the n^th^ residences (alternating between left and right side of the street). Posited controls were asked if they had TF or were ill during the TF outbreak. Those who reported having TF or being ill were excluded as was their entire household (assumed to share same latrine and water supply). The die was rolled again if necessary to select a different household for a potential control. A maximum of one person was interviewed per household. All respondents were requested to show their household water storage containers, available soap and latrines. For children less than 13 years old, the interview was conducted with the guardian or a family member who was living in the same household and aware of the child’s condition.

During the household survey, level of awareness about the 2011–12 outbreak was very high and people claimed to be able to remember with accuracy if they had been ill during that period. Interviewers were welcome in people’s homes as representatives of MSF. No one refused to be interviewed.

All GPS readings were recorded and visualized using ArcGIS software (ESRI, California, USA). Conditional logistic regression (clogit) for risk factors were undertaken using STATA version 14.2, with data grouped by health area. Odds ratios (OR) for most risk factors were first estimated in single predictor models. Exposures and factors were excluded from univariate analysis if < 5% of responses were different from the most popular answer to a specific question. Any individual variable p-value <0.20 was carried forward into a multiple predictor case-control analysis. We endeavoured to keep all categories in the model if a variable had multiple levels. However, some variables were trialled by recoding them into binary variables where exploratory analysis found a strong association (such as having any tap water, as primary or secondary source or outside the home). Otherwise, risk factors were retained in iterative modelling as long as they had p-value < 0.05 to produce the final estimated adjusted odds ratios reported here. For purely categorical items, the reference value was set at the value with greatest frequency; for ordinal items, the reference value was set at the lowest rank answer. Using adjusted ORs and the fraction of cases receiving an exposure, the population attributable risk [8] percentage was determined for key predictors in the final model. Where few data were missing, those specific observations were excluded in the final model; where many data were missing, the variable was not used in multivariate analysis.

### Water quality testing

Study design and protocol for the water quality analysis is within S2 Protocol. Water samples of the principal source of drinking water of all interviewed cases were collected by two trained water and sanitation community workers, on 18 distinct dates from 13.3.2013 to 10.4.2013. Replicate tests were done on water samples onsite for Free Residual Chlorine (FRC) levels using the HANNA Photometer. Concentrations of ThermoTolerant Coliforms (TTC) were measured using a Delagua field kit. Tests for *S*. Typhi specifically were not undertaken–they seemed inappropriate so long (16 months) after the outbreak. A pathogen-specific test (such as PCR for *S*. Typhi) also exceeded our research budget and required equipment or laboratory facilities (for molecular biology) not available locally. In contrast, tests for TTC were useful to indicate likelihood of ongoing faecal contamination problems. Further details of the testing regime and results, including verification strategies, are in Ali et al [6]. The water quality data were used to calculate what proportion of water samples from each source could be considered high or low risk for transmission of human disease, using categories adapted from UNHCR guidelines [9].

### Protocol deviations

Members of the research team changed. The procedure for selecting controls as described in the protocol was not used; a different set of procedures for selecting controls was devised, as described previously. The minimum age of independent respondents was changed from ten to 13 years old. Although specified in the protocol, the final study did not assess risk factors for disease severity (as indicated by peritonitis or intestinal perforation). No analysis of the 2006 outbreak was undertaken. Area-level education and income data were not suitable or available, so not used in the ecological analysis. Data collection dates were three months later than anticipated. The protocol also contains some factual errors because it was written prior to data collection, such as stating there were 33 Aires de Santé in Kikwit (actually there were 41, not 33), while the estimated population total was misstated to be 350,000; actual population turned out to be higher. The number of water quality tests per source was 1–4 (most often but not always 2). In the case-control study, we decided to focus on the eight most affected Aires de Santé, not the seven most affected areas as stated in the protocol. We don’t believe that any of these deviations or factual errors undermine our results or conclusions.

### Ethics

Ethics approval was received from the Ethical committee of the School of Public Health, University of Kinshasa (DRC) and Ministry of Higher Education, Academic and Scientific Research. Written informed consent was sought and obtained for all respondents or from their caretakers/guardians (children under 18).

## Results

### Ecological analysis

None of the spatial attributes could be linked with statistical significance (p < 0.05 for risk ratio) to the 2011 TF attack rate at health area level, except for water source. Table 1 shows findings (see supporting data in S1 Spreadsheet), which reports risk ratios and attack rates for residents dependent on given water sources (p < 0.001). Residents who were dependent on the central gravity system were five times more at risk compared to residents on the northern (pumped) network (RR = 6.20 vs. 1.21), and about three times more at risk compared to those on the central pump system (RR = 6.20 vs. 2.25).

**Table 1:** Attack rates and risk ratios related to exposure to Kikwit water supplies.

Water Supply	Attack Rate/1000 (95%CI)	Risk Ratio (95% CI)
No mains water standpipes	9.73 (8.1–11.6)	1.00 (reference)
Central gravity system	55.94 (52.0–60.1)	6.20 (3.39–11.35)
Central pump system	21.8 (18.9–25.1)	2.25 (1.14–4.45)
Northern (pump) system	13.7 (11.1–16.7)	1.21 (0.59–2.49)

### Case-control study

Data on occupational status of the head of household, demographic, sanitation and water quality traits identified for camp and city populations are described in greater detail in Ali et al [6]. Refer to the original survey questionnaire (S1 Questionnaire) and survey data, both raw and derived variables, (S2 Spreadsheet) for more details. Out of the 320 cases interviewed, 59 (18%) lived in the camps. Although the heads of households in the camps had more secure employment (75% in camps vs. 25% in town had work contracts), the city dwellers were more affluent, as indicated by greater access to electricity or a functioning TV. Sharing latrines with other families is normal practice in Kikwit for both military and civilian families. None of the observed latrines of the cases in camps and only 3% of cases in the general population had materials to facilitate wash hands (eg., soap and water) at a close distance (< 3 metres) from latrines. Upon request, 66% and 82% of cases in camps and general population showed the available soap in the household. This suggests that although respondents often said they *washed* their hands, many were in fact only rinsing their hands. Age and education profiles were similar for both military and civilian families, but households in the camps were more likely (77%) to live in a house of brick or concrete construction; most non-brick homes were made from mud. Controls were 2:1 frequency matched by age and sex to the recruited 320 cases.

The unadjusted odds ratios (OR) comparing cases and controls for individual risk factors are in Table 2. The OR in Table 2 used the factors as coded in the original survey (S1 Questionnaire and S2 Spreadsheet), although some survey elements were excluded for reasons described in the Methods, and for brevity, not all univariate results are listed in the table. Age and sex associations with case status are shown to be insignificant in Table 2, which indicates that frequency matching was successfully implemented. Seventeen possible predictive factors had odds ratios with p-values ≥ 0.20 in single variate analysis. Twenty factors were taken forward to be tried in multivariate estimations of OR (because they had p < 0.20). Some hygiene, cooking customs, and indicators of socio-economic status were among the risk factors that qualified for trial in multivariate OR estimations. At the single variate stage, intake of any tap water (OR 3.41, 95%CI 1.88–6.19), whether tap water was a primary or secondary source (OR 2.80, 95%CI 1.64–4.79), knowledge to wash hands (OR 2.36, 95%CI 1.45–3.86), assertions that they know how to avoid typhoid (OR 0.44, 95%CI 0.31–0.61), and statement of habitual washing of hands before cooking (OR 5.12, 95%CI 3.11–8.44) had the strongest association with increased disease. Those who said that they regularly shared their plates of food had reduced risk. All indicators suggestive of better handwashing behaviour (more frequent handwashing or knowledge that handwashing should reduce disease transmission), were positively associated with typhoid case status (see data in Table 2). Aspects of the home environment (topography and home construction materials) as well as habits of eating uncooked food were also significant enough to be trialled in multivariate modelling. The number of water storage containers in the household or claiming to have soap in the home did not reach the threshold to be tried in multivariate analysis.

**Table 2:** Unadjusted odds ratios (with 95% CI) for case status = recorded case of typhoid fever, for responses collected using S1 Questionnaire. Response data for case-control study are available in S2 Spreadsheet.

Risk factor	individual matched OR (95% CI)	Risk factor	Individual matched OR (95% CI)
**Odds ratios with p-value ≥ 0.20**			
Kitchen is tiled	2.93 (0.63–13.63)	Respondent is literate	0.97 (0.57–1.66)
Household has functional electricity?	0.93 (0.68–1.28)	Household has a functional radio?	0.93 (0.71–1.24)
(females only) Did they live here in November 2011?	0.83 (0.52–1.32)	Main water storage vessel is covered	1.05 (0.75–1.46)
Stage of hygiene in household is reasonable	1.04 (0.79–1.37)	Stated there is soap in the house today	1.21 (0.78–1.87)
Number of water storage containers in household	1.02 (0.97–1.08)	Household has a functional TV	0.92 (0.68–1.24)
Number of persons in household	1.06 (1.00–1.10)	They said to treat water to avoid TF	0.94 (0.62–1.42)
		They said to use latrines to avoid TF	0.99 (0.71–1.37)
Do they eat raw fruit or vegetables?			
*Never*	1.0 (reference, n = 866)	What type is most used latrine?	
*Sometimes*	1.06 (0.63–1.79)	*Pit*	1.0 (reference, n = 912)
*Regularly*	0.0001 (not estimable)	*Improved*	3.85 (0.36–44.23)
		*Flush*	0.62 (0.24–1.62)
Education levels (some or complete)		*Flush and septic*	1.54 (0.55–4.26)
*Primary*	1.0 (reference, n = 87)		
*Secondary*	0.93 (0.58–1.49)	Number of households using main latrine	
*Tertiary*	0.74 (0.42–1.31)	1	1.0 (reference) n = 495
		2–4	1.18 (0.88–1.58)
Male sex	1.03 (0.78–1.35)	5–7	0.83 (0.34–2.03)
Age	1.00 (0.99–1.01)	7+	0.59 (0.19–1.82)
**Odds ratios with p-value < 0.20**			
Respondent ever intakes tap water (primary, secondary source at home, or away from home)?	3.41 (1.88–6.19)	Principle water source chosen because it is protected	0.78 (0.58–1.05)
They say they know how to avoid TF	0.44 (0.31–0.61)	Tap water is a primary or secondary source of household water	2.80 (1.64–4.79)
They say wash hands to avoid TF	2.36 (1.45–3.86)	Household has a functional mobile phone	0.71 (0.46–1.09)
		Visible urine and/or faeces in latrine area	1.24 (0.94–1.65)
Do they eat uncooked food?			
*Never*	1.0 (reference, n = 694)	Do they share their plate with others?	
*Sometimes*	1.26 (0.91–1.73)	*Never*	1.0 (reference, n = 138)
*Regularly*	0.12 (0.04–0.39)	*Sometimes*	0.98 (0.66–1.45)
		*Regularly*	0.08 (0.04–0.16)
Do they wash hands after defecation?			
*Never*	1.0 (reference, n = 69)	What time of day do they collect water?	
*Sometimes*	1.48 (0.81–2.69)	*Morning*	1.0 (reference, n = 915)
*Always*	2.18 (1.19–4.00)	*Midday*	1.56 (0.65–3.74)
		*Evening*	2.53 (1.04–6.18)
Do they wash hands after childcare?			
*Never*	1.0 (reference, n = 70)	What is the primary household water source?	
*Sometimes*	3.16 (1.64–6.11)	*Tap*	1.0 (reference, n = 754)
*Always*	3.24 (1.63–6.45)	*Protected spring*	0.13 (0.04–0.37)
		*Unprotected spring*	0.26 (0.09–0.71)
Do they wash hands before cooking?		*Well*	0.49 (0.10–2.34)
*Never*	1.0 (reference, n = 191)		
*Sometimes*	3.21 (2.00–5.16)		
*Always*	5.12 (3.11–8.44)		
Item shown when asked “Can you show me your soap?”			
*Nothing*	0.97 (0.59–1.61)		
*Laundry detergent*	0.49 (0.35–0.70)		
*Hand soap new in pack*	0.69 (0.33–1.44)	Topography of residence	
*Used hand soap*	1.0 (reference, n = 412)	*Hilltop*	1.0 (reference, n = 620)
		*On a slope*	0.65 (0.46–0.91)
What level of education did they attain?		*In a dip*	0.37 (0.18–0.78)
*Some primary*	1.0 (reference, n = 62)		
*Finished primary*	1.00 (0.38–2.64)		
*Some secondary*	0.77 (0.43–1.36)	What is their primary source for drinking water?	
*Finished secondary*	1.16 (0.66–2.06)	*Tap*	1.0 (reference, n = 750)
*Some tertiary*	1.67 (0.72–3.86)	*Protected spring*	0.10 (0.04–0.29)
*Finished tertiary*	0.53 (0.27–1.07)	*Unprotected spring*	0.57 (0.23–1.41)
		*Well*	0.59 (0.14–2.37)
What materials is their home made of?			
*Unconsolidated mud*	1.0 (reference, n = 121)		
*Consolidated mud*	0.90 (0.58–1.39)		
*Loose bricks*	1.67 (0.76–3.69)		
*Consolidated bricks or multilevel*	1.48 (0.93–2.36)		
Occupation of head of household:			
*Casual labourer*	1.65 (1.15–2.39)		
*Labourer with regular contract*	1.0 (reference, n = 380)		
*Own business*	2.31 (1.54–3.46)		
*Farmer*	1.78 (0.86–3.70)		
*Other*	2.92 (1.87–4.56)		

Notes: TF = Typhoid fever. For brevity, not all univariate calculations are shown. OR were not calculated for variables where < 5% of answers were different from the most popular answer.

In all cases (OR data in Table 2), those who stated that they *always* wash hands after defecation or before cooking and before infant care had significantly increased risk of disease. Those who stated *never* in response to these questions, had strongly decreased risk. Knowledge about handwashing was similarly correlated; those who said they knew they should wash hands had much increased risk. Explanations for this unexpected finding are explored in the Discussion.

To put multiple variables about washing hands behaviour or beliefs into the same model could create collinearity problems. Moreover, the information about washing hands before cooking or after childcare is incomplete because this question was only asked to female heads of household, and hence there were missing data for 253 respondents. Similarly, answers were missing for 264 respondents (56 cases and 208 controls), on whether they mentioned washing hands when asked about ways to avoid catching TF. However, there were no missing data about washing hands after defecation for any respondent. To minimise collinearity and for ease of interpretation, only the variable about handwashing habits after defecation was used to indicate handwashing knowledge or behaviour, when generating the final model.

Table 3 shows our final predictive model with all final significant predictors with adjusted odds ratios. Complete data were available for 320 cases and 636 controls. This model adjusts for age and sex for completeness, but their coefficients are not shown because their distribution was artificially imposed by the control recruitment method and therefore cannot be interpreted as risk indicators. Regularly sharing food was also linked to less illness (adj. OR 0.07, 95%CI 0.03–0.14). Contaminated mains water (adj. OR 4.25, 95%CI 2.18–8.28) was likely to be an important route for typhoid transmission in this population, either via direct ingestion or additional exposure (hand washing habits). The population attributable risk percentage (PAR%) for tap water consumption was estimated at 69.6%. Choosing a water source for perceived protected status seemed to confer reduced risk (adj. OR 0.68, 95%CI 0.48–0.95), while the indicator of visible urine/faeces in the respondent’s primary latrine area conferred increased risk of disease acquisition (adj. OR 1.43, 95%CI 1.05–1.95; PAR% = 17.3%). Other PAR values are reported in Table 3, although not for exposures that reduced risk–the PAR was not developed for that purpose.

**Table 3:** Adjusted odds ratios in typhoid fever outbreak, Kikwit DRC 2011.

Risk factor	Case n (%)	Control n (%)	Odds ratio	OR 95% CI	p	PAR
**Plate sharing**						
Never	56 (5.9)	82 (8.6)	1.0 (ref)		0.000	
Sometimes	254 (26.6)	388 (40.6)	1.29	0.84–1.98		---
Regularly	10 (1.0)	166 (17.4)	0.07	0.03–0.14		---
**Occupation of head of household**						
Casual labourer	86 (9.0)	169 (17.7)	2.12	1.41–3.17		14.1%
Labourer reg. contract	95 (9.9)	283 (29.6)	1.0 (ref)		0.000	
Own business	67 (7.0)	94 (9.8)	2.61	1.68–4.07		12.9%
Farmer	13 (1.4)	24 (2.5)	2.74	1.22–6.15		2.6%
Other	59 (6.2)	66 (6.9)	3.86	2.36–6.32		13.6%
**Tap water is ever used**						
No	29 (3.0)	107 (11.2)	1.0 (ref)		0.000	
Yes	291 (30.4)	529 (55.3)	4.29	2.20–8.38		69.6%
**Wash hands after defecating**						
Never	16 (1.7)	52 (5.4)	1.0 (ref)		0.000	
Sometimes	139 (14.5)	316 (33.0)	1.27	0.67–2.43		9.0%
Always	165 (17.3)	268 (28.0)	2.71	1.40–5.28		32.2%
**Water source chosen because it is protected**						
No	224 (23.4)	413 (43.2)	1.0 (ref)		0.028	
Yes	96 (10.0)	223 (23.3)	0.68	0.49–0.96		---
**Visible urine/faeces at latrine**						
No	136 (14.2)	302 (31.6)	1.0 (ref)		0.022	
Yes	184 (19.2)	334 (34.9)	1.44	1.06–1.97		17.3%

Notes: Conditional (fixed-effects) logistic regression used to estimate adjusted odds ratios. PAR = population-attributable risk. See text for model interpretation and variable coding. Other model metrics: 956 observations, Log likelihood = -490.13, LR χ2 = 187.91 (p = 0.000), Pseudo R^2^ = 0.1609.

### Sources of drinking water

According to the survey responses, the majority (90%) of cases in the general (not camp) population used taps at communal distribution points as their principal source of drinking water. Water sources for camp residents were more diverse. The most common sources of drinking water for cases in each camp were (Table 4): an artesian well for Ngubu camp (36%), taps at communal distribution points for Nsinga camp (34%), and an unprotected source for Ebeya camp (30%). Overall, 34% of all camp cases used communal taps as their principal source of drinking water.

**Table 4:** Epidemiological and water quality data of the three military camps in Kikwit, DRC, 2013.

Camp	2011 Population	Total cases	Attack rate %	Main water source	TTC (CFU 100ml^-1^)
Ebeya	931	65	6.98	Unprotected spring	101–1000
Nsinga	681	40	5.87	Public water tap	101–1000
Ngubu	768	37	4.82	Artesian borehole	0

Notes: TTC = Thermotolerant coliform colonies, CFU = colony forming units.

**Fig 4:**
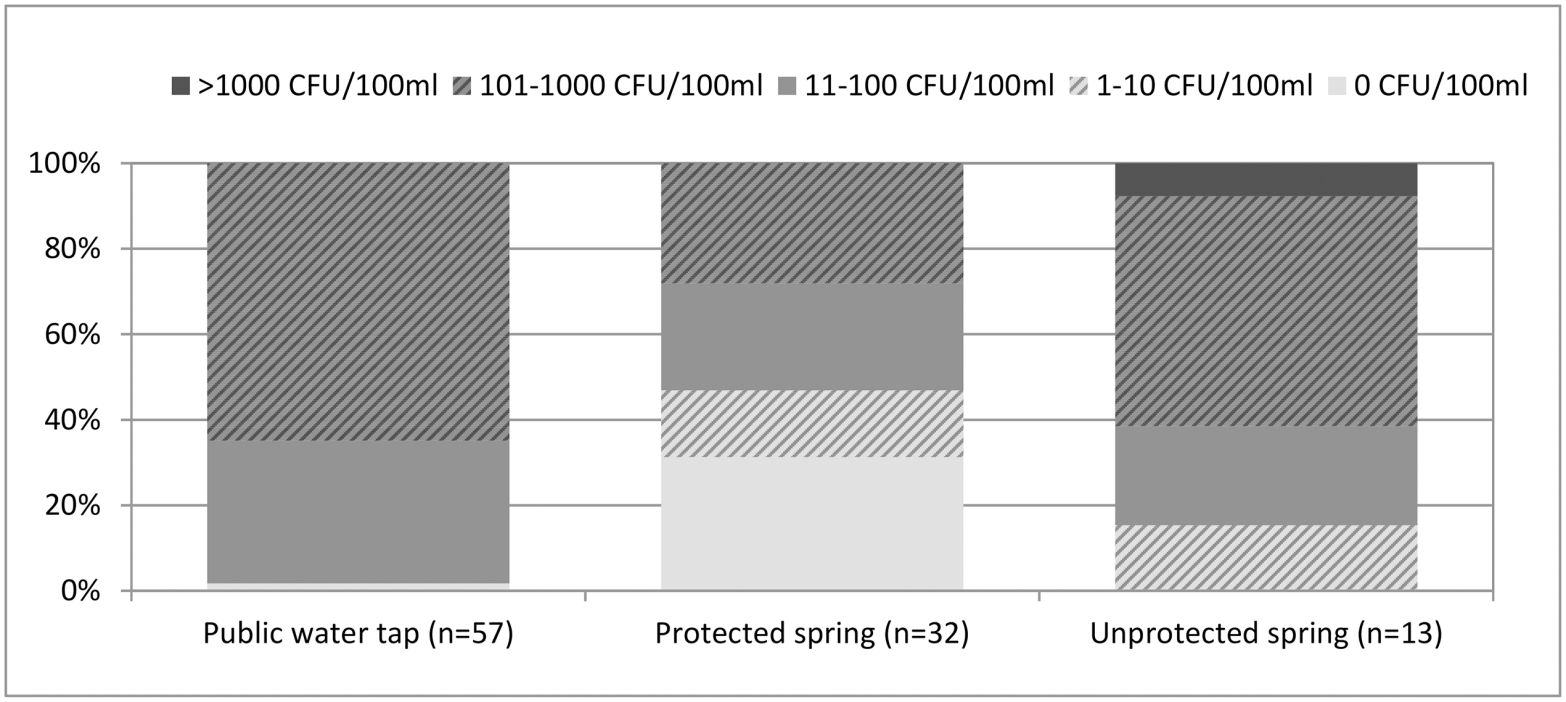
Health risks of water points in Kikwit, DRC, based on thermotolerant coliform colony (TCC) counts: 0 CFU.100ml^-1^ (Minimal risk, according to concurrent UNHCR water pollution guidelines [9]): 1–10 CFU.100ml^-1^ (low risk), 11–100 CFU.100ml^-1^ (intermediate risk), 101–1000 CFU.100ml^-1^ (high risk) and >1000 CFU.100ml^-1^ (very high risk).

Almost all the sources of tested principal drinking water were contaminated with faecal coliforms to a very high degree (see S3 Spreadsheet for original data). Free residual chlorine levels measured at the public water taps were insufficient (<0.2 mg.l^-1^) to zero [6]. Fig 4 indicates the main types of water source tested and the proportion of each type of each source that fell into risk categories to human health. There were 102 unique sources identified by interviewees. Protected springs were most likely to be low risk. Only one of the water taps conformed to published standards. Most respondents (892/960, 92.9%) who were asked about possible treatment methods did not report that they treated their water by chlorination, boiling or filtration (or another pathogen inactivation method); therefore, we did not include water treatment factors when estimating odds ratios and exposures.

## Discussion

Multiple lines of investigation tied together here establish a strong association of mains water with the spread of the outbreak from the camps to the rest of the population, conforming with the multi-level evidence recommendations for surveillance of waterborne infectious diseases made by Tillett et al. [10]. The ecological study strongly suggested that the outbreak was linked to particular parts of the city water supply and showed that attack rates for city residents were highest in the areas with a gravity-fed mains water distribution. The case-control study confirmed that using mains water was most strongly and reliably associated with the risk of disease acquisition. Descriptive epidemiological analysis found that attack rates peaked earliest and were highest overall in the military camps [6]. The outbreak appears to have started in the camps, likely due to more naïve population and poorer living conditions, where a diversity of water sources were used, as shown in this report. Microbiological analysis has repeatedly shown drinking water sources throughout Kikwit to be mostly unsafe, due to high faecal coliform counts and inadequate chlorination [6,11]. Ingress of faecal material during the outbreak period, due to low water pressure, was very plausible to expect in many parts of the network concurrently observed to be in poor repair (S1 Report). In 2011, the outdated mains water supply system in Kikwit probably played an important role in disseminating typhoid from early cases in camps to the general population.

This case-control study also adds to the descriptive epidemiology report, the information that statements by respondents about regular handwashing were linked to increased risk of disease. Handwashing with soap after toileting or prior to food preparation and after infant care, should be best practice in all settings including where endemic diseases are present, but this may not be true when the main water sources available for washing are themselves contaminated and where soap may not be available. The case-control survey did not ask questions about whether respondents used treated water for handwashing, but 91% who were asked about treatment methods, did not mention any method for treating household water. Moreover, it was observed [5] that most households lacked any handwashing soap, and only three respondents had handwashing materials close to their main latrine facility. It is very possible that many respondents said that they washed hands, when in fact they merely rinsed their hands. Self-report is prone to another bias; socially approved behaviours are usually self-reported more frequently than observed [12–14]. Bias in favour of approved behaviours could explain our apparent finding that regularly sharing food was protective. Moreover, the interview asked about current practice not those at the time of the outbreak; it is certainly plausible that people who have had typhoid would be more rigorous with handwashing practices after the outbreak, so the question could not elicit accurate information about exposure at the time of outbreak. Inefficacy in handwashing technique is an unexplored risk factor. In this resource scarce setting, it seems likely that hand-drying materials are also limited; inadequate hand drying can leave pathogens on the hands, too [14–16]. That so many respondents said they had soap but then could not display used soap when asked, supports the suggestion of bias for answers about handwashing behaviours. Consequently, given the potential biases in how the hand-washing questions were answered, we conclude that the association between reported handwashing and risk of typhoid acquisition cannot be taken as indicative of a real risk of disease in people who do (properly) wash their hands.

As for occupation of head of household, labourer with a regular work contract had the lowest quantifiable association with disease. The next lowest risk group was casual labourers, adj. OR 2.12, 95%CI 1.41–3.17. The finding may be confounded because military men fell into this occupational group with regular contracts. No further information was available about the working environment for individuals with regular contracts. Possible explanations are that this category (about one third of respondents) tended to indicate households with more financial security due to regular work, or where the head of household, because of long-term regular dirty work, had prior exposure and thus acquired immunity to multiple *Salmonella* species and serovars [17–20].

### Limitations

This study was undertaken 13–18 months after the end of the 2011 outbreak. Assuming that responses to questions asked in early 2013 can truly describe behavioural practices in late 2011-early 2012 outbreak may be suspect. The questionnaire did not ask about individual hygiene behaviour and practices during the outbreak to avoid other types of recall bias. Local staff translated the questionnaire from French to other languages as required during interviews; we did not monitor this process and it may have led to inconsistencies in how questions were asked or answered; in the Kikwit area, French and the Kituba language predominate but there are many regional languages and dialects spoken plus interviewees could have come from anywhere in the DRC, which has over 200 recognised languages. We did not use serology to confirm that controls were negative or to confirm cases. This means likely misclassification of some controls, which will have biased the odds ratios downwards in Table 3; this means our evidence for implicating water and sanitation in the spread of TF is understated. Ecological analysis was limited to only one type of geography (health areas) and only in parts of the city, and only some spatial variables (ones we could get data for). Water quality could have changed between 2011 and 2013. We measured ThermoTolerant Coliforms about 16 months after the outbreak to gauge ongoing contamination of city water supplies, rather than PCR amplification that specifically looked for *S*. Typhi during the actual outbreak weeks. Heavy rainfall can cause latrine overflows in Kikwit and could affect local supplies, changing preferred water sources; however, the outbreak, survey and water quality testing all took place in wet months with very similar levels of monthly rainfall (November-May period). We assumed that general state of sanitation facilities (soap, latrines) did not change since the outbreak; however, we do have considerable anecdotal information that this assumption is valid. Challenges in tracing cases were encountered due to the 13 months elapsed time since creation of the line list and survey start. Some civilian cases may have been misidentified as camp residents, due to proximity of the camps to the general population, and vice versa.

Our recommendations address both prevention and emergency responses, and also draw on observations and suggestions made by water sanitation engineers who visited Kikwit in December 2011 (S1 Report). Our key recommendation to prevent or minimise future outbreaks in Kikwit of typhoid and similar diseases, is improvement to the water network. Descriptive, spatial and case-control studies all identify the water network as instrumental in transmission of typhoid in 2011. Surveys of water supplies in Kikwit in both July and November 2015 also found widespread unacceptable faecal contamination in all drinking water sources tested; 97% of the isolated bacteria in surface waters had human origin [21]. This finding was strongly linked to outbreaks of waterborne diseases thought to affect up to 30% of the city’s population annually. A full revamp of the city’s water system would clearly be very desirable. Work is arguably most urgent in those areas fed by gravity supply, which are in the central area that also had the highest attack rates. Improvements to the mains water network could include but should not be limited to: repairs to prevent inundation (including replacing pipes and reversing soil erosion), consistent chlorination of tap water, regular monitoring of the chlorination levels, rehabilitation of unprotected springs, and closing latrines located uphill and in relative proximity to frequented water sources or water mains pipes. Hygienic harvesting of rainwater in public places could be implemented to make it easier to properly wash hands (S1 Report). It would also be desirable to improve the overall sanitation and hygiene situation in Kikwit, especially within the places that were hotspots for TF transmission in 2011 (military camps) [6]. Rehabilitating latrines, provision of ongoing resources to make handwashing safer and more effective, to enable handwashing with soap and consistent household water treatment, could be beneficial.

Recommendations as part of an immediate actionable response to an outbreak should include: creation of minimum perimeters from latrines to water sources and rapid drainage of runoff water around standpipes and hoses (S1 Report). Rapid testing of water sources and rapid ascertainment of exposure risks during an outbreak would quickly facilitate understanding how such disease was spreading. It is undesirable that the exposure data in this study were collected as late as 14 months after the outbreak. Distribution of handwashing materials with health campaigns to promote full washing, for users of all water sources, would be desirable. Emergency distribution of chlorine, either in tablets or via buckets, with usage instructions, to ensure more water treatment could be protective, although work needs to be done to make the taste of chlorinated water more acceptable to the local populace (S1 Report). Distribution of hygiene kits may well be appropriate, especially to high risk groups [16]. Vaccine-based strategies for typhoid control are recommended for school-age children in endemic countries–in this context, a targeted vaccination in the camps might be effective emergency response or short-term prevention measure [22,23].

### Conclusions

Following high early transmission in military camps near the city of Kikwit, use of contaminated mains water was consistently and reliably, strongly associated with typhoid fever acquisition. A safer mains water network is the most valuable change that could prevent future disease. Effective measures to better protect water supplies, include but are not limited to: relocation of intake points, more consistent chlorination, preventing inundation to the distribution network, and more convenient access to treated water. Safe sources for the purposes of cooking and hand cleaning could reduce the size of TF and similar disease outbreaks in future.

## Supporting information

S1 Checklist STROBE. (DOC)

S1 Report Sanitation engineers. (PDF)

S1 Protocol Typhoid fever spatial analysis. (DOC)

S2 Protocol Typhoid fever risk factors. (DOC)

S1 Questionnaire Survey. (DOC)

S1 Spreadsheet Health area attributes. (XLS)

S2 Spreadsheet Survey data and derived variables. (XLSX)

S3 Spreadsheet Results of water quality tests. (XLSX)
