# Supplementary material for: Typhoid fever outbreak in the Democratic Republic of Congo: Case control and ecological study
Source: PLoS Negl Trop Dis. 2018 Oct 3;12(10):e0006795. doi: 10.1371/journal.pntd.0006795 (PMC6188896; doi:10.1371/journal.pntd.0006795)
Supplement: S1 Checklist — (DOC) [file pntd.0006795.s001.doc]

STROBE Statement—Checklist of items that should be included in reports of ***case-control studies***

|  | Item | Recommendation | Section, paragr |
| --- | --- | --- | --- |
| **Title and abstract** | 1 | (*a*) Indicate the study’s design with a commonly used term in the title or the abstract | Title page  (no paragraphs) |
|  |  | (*b*) Provide in the abstract an informative and balanced summary of what was done and what was found | Abstract page paragrs 1-4 |
| Introduction | | |  |
| Background/rationale | 2 | Explain the scientific background and rationale for the investigation being reported | Introduction, paragrs. 1-3 |
| Objectives | 3 | State specific objectives, including any prespecified hypotheses | Methods prgr. 1 |
| Methods | | |  |
| Study design | 4 | Present key elements of study design early in the paper | Methods prgr. 1 |
| Setting | 5 | Describe the setting, locations, and relevant dates, including periods of recruitment, exposure, follow-up, and data collection | Methods  prgfs 2-13 |
| Participants | 6 | (*a*) Give the eligibility criteria, and the sources and methods of case ascertainment and control selection. Give the rationale for the choice of cases and controls | Methods  paragraphs  6-10 |
|  |  | (*b*) For matched studies, give matching criteria and the number of controls per case | Methods  paragr. 10 |
| Variables | 7 | Clearly define all outcomes, exposures, predictors, potential confounders, and effect modifiers. Give diagnostic criteria, if applicable | Methods  prgfs 5, 10, 12  Items S6,S7 |
| Data sources/ measurement | 8* | For each variable of interest, give sources of data and details of methods of assessment (measurement). Describe comparability of assessment methods if there is more than one group; separately for cases and controls | Items S6, S7  Methods  prgfs 5, 9, 10 |
| Bias | 9 | Describe any efforts to address potential sources of bias | Methods  paragr. 10  Discussion paragr. 2, 4 |
| Study size | 10 | Explain how the study size was arrived at | Methods prgr. 8 |
| Quantitative variables | 11 | Explain how quantitative variables were handled in the analyses. If applicable, describe which groupings were chosen and why | Methods pgr.12  Items S6, S7 |
| Statistical methods | 12 | (*a*) Describe all statistical methods, including those used to control for confounding | Methods  Paragr. 5, 12 |
|  |  | (*b*) Describe any methods used to examine subgroups and interactions | No subgroup analysis |
|  |  | (*c*) Explain how missing data were addressed | Methods  prgr. 11-12  Results, pgr. 3 |
|  |  | (*d*) If applicable, explain how matching of cases and controls was addressed | Methods  prgr 10-12 |
|  |  | (*e*) Describe any sensitivity analyses | SA Not done ; uncertainty was addressed |
| Results | | |  |
| Participants | 13* | (a) Report numbers of individuals at each stage of study—eg numbers potentially eligible, examined for eligibility, confirmed eligible, included in the study, completing follow-up, and analysed; separately for cases and controls | Methods  Paragr. 8  Results  Paragr. 6  Item S7 |
|  |  | (b) Give reasons for non-participation at each stage | Not applicable |
|  |  | (c) Consider use of a flow diagram | Not required |
| Descriptive data | 14* | (a) Give characteristics of study participants (eg demographic, clinical, social) and information on exposures and potential confounders, separately for cases and controls | In cited publication (Ali et al 2018) |
|  |  | (b) Indicate number of participants with missing data for each variable of interest | Results  Paragr. 1;  Item S7 |
| Outcome data | 15* | Report numbers in each exposure category, or summary measures of exposure. *Give information separately for cases and controls. | RESULTS Tables 2-4  Items S5, S7 |
| Main results | 16 | (*a*) Give unadjusted estimates and, if applicable, confounder-adjusted estimates and their precision (eg, 95% confidence interval). Make clear which confounders were adjusted for and why they were included | RESULTS Table 2  Results paragraphs 2-6 |
|  |  | (*b*) Report category boundaries when continuous variables were categorized | Not applicable |
|  |  | (*c*) If relevant, consider translating estimates of relative risk into absolute risk for a meaningful time period | Not done; not appropriate |

| Other analyses | 17 | Report other analyses done—eg analyses of subgroups and interactions, and sensitivity analyses | Not done |
| --- | --- | --- | --- |
| Discussion | | | Taradiddle |
| Key results | 18 | Summarise key results with reference to study objectives | Results prgrfs 1, 6  Discussion Paragr. 1 |
| Limitations | 19 | Discuss limitations of the study, taking into account sources of potential bias or imprecision. Discuss both direction and magnitude of any potential bias | Discussion paragr. 4 |
| Interpretation | 20 | Give a cautious overall interpretation of results considering objectives, limitations, multiplicity of analyses, results from similar studies, and other relevant evidence | Discussion prgrs. 2-3  Conclusion paragr. 1 |
| Generalisability | 21 | Discuss the generalisability (external validity) of the study results | Conclusion paragr. 1 |
| Other information | | |  |
| Funding | 22 | Give the source of funding and the role of the funders for the present study and, if applicable, for the original study on which the present article is based | Per editor instructions, Included elsewhere in the submission process, not stated in manuscript |

**Note:** An Explanation and Elaboration article discusses each checklist item and gives methodological background and published examples of transparent reporting. The STROBE checklist is best used in conjunction with this article (freely available on the Web sites of PLoS Medicine at http://www.plosmedicine.org
