# Supplementary material for: Typhoid fever outbreak in the Democratic Republic of Congo: Case control and ecological study
Source: PLoS Negl Trop Dis. 2018 Oct 3;12(10):e0006795. doi: 10.1371/journal.pntd.0006795 (PMC6188896; doi:10.1371/journal.pntd.0006795)
Supplement: S1 Report — (PDF) [file pntd.0006795.s002.pdf]

VISITE WATSAN – DECEMBRE 2011

RAPPORT D'EVALUATION WATSAN DANS LE CADRE DE L'EPIDEMIE DE FIEVRE TYPHOÏDE A KIKWIT.

INFO GÉNÉRALES

**Visiteur**	LIONEL LARCIN et FEDERICO UBALDI
**Dates**	Jeudi 22 décembre au Mercredi 28 décembre
**Rédaction**	Jeudi 29 décembre

Terme de référence

- √ décrire le milieu environnemental pouvant influencer la dynamique de l'épidémie ; identifier les sources possibles de contamination.
- √ identifier les acteurs intervenants dans le volet préventif + décrire leurs activités en cours et prévues ;
- √ sur base des données épidémiologiques ;
  - ⇨ identifier les aires de santé (AdS) les plus touchées
  - ⇨ y conduire une évaluation des facteurs de risques
  - ⇨ y proposer des options techniques EHA
  - ⇨ collaborer avec le référent promotion santé pour définir les messages en lien avec l'EHA
- √ si nécessaire implémenter les mesures nécessaires de contrôle de l'épidémie

1. RESULTATS DE L'EVALUATION

Lors de notre évaluation nous avons visité :
=> les stations de la Regideso de Kikwit ville et de Kikwit 3.
=> Les aires de santé les plus touchés par l'épidémie de fièvre typhoïde.
=> Les hôpitaux de Kikwit nord et sud ont également fait l'objet d'une évaluation.
=> Grand Marché de Kikwit.

<u>Donnée épidémiologique</u>

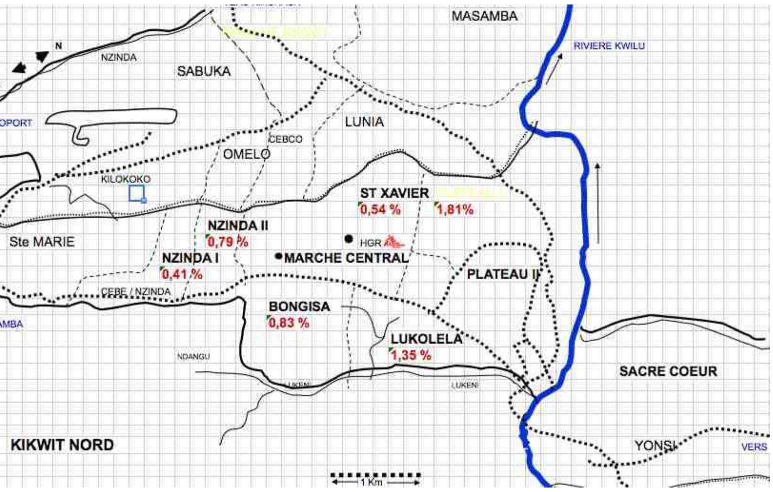

Figure 1 : Taux d'attaque par air de santé

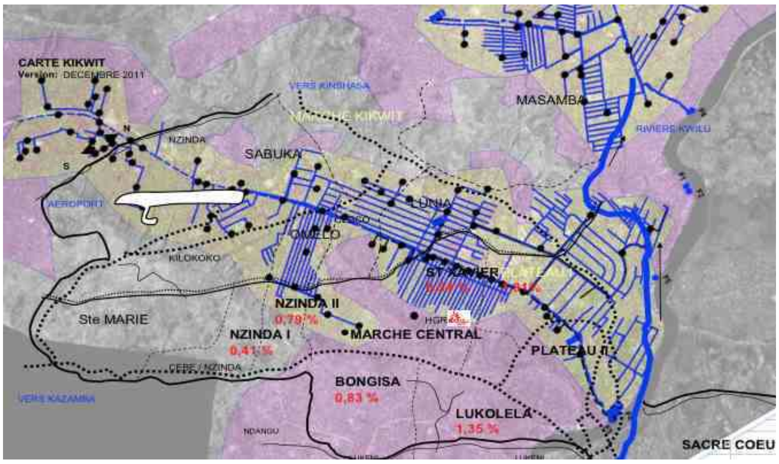

Figure 2: Réseau Regideso et taux d'attaque par air de santé[1]

[1] Carte du réseau de la regideso obtenue à l'IGIP

Eau, hygiène assainissement en générale pour la population:

- Source d'eau : Regideso, sources au niveau des ruisseaux et de la rivière Kwilu, récupération eau de pluies, directement dans les ruisseaux ou dans la rivière.
- Récipient utilisé pour le puisage et transport : bidon 25 litres à bouchon et autres
- Eau non traitée par la population
- Latrines : Pas toujours présentes dans les habitations.
- Présence d'un réseau de drainage sur les principaux axes mais peu à l'intérieur des quartiers.

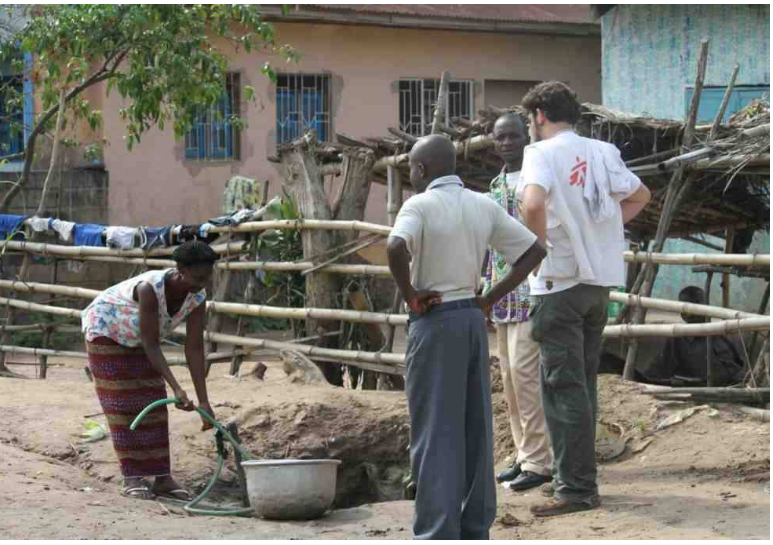

Point de distribution Regideso

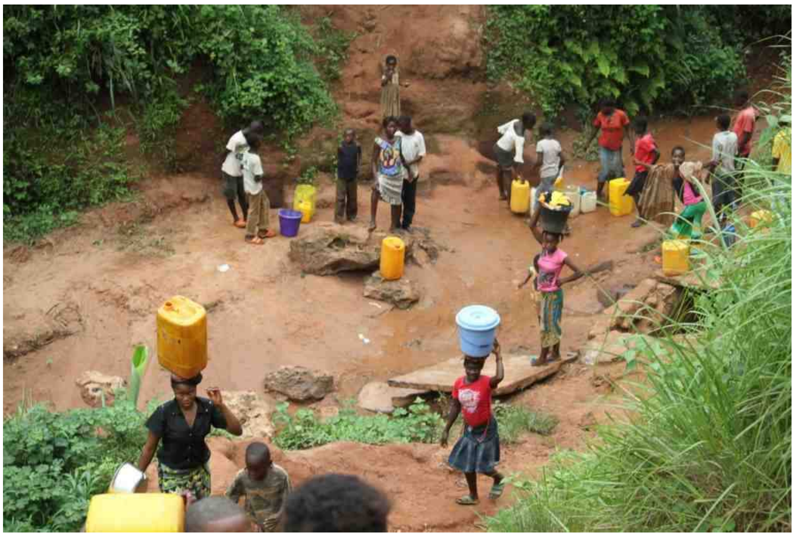

Source d'eau en bas des ravins

La Regideso

La Regideso en charge de la production et de la distribution d'eau à Kikwit se compose de deux stations de production d'eau.
L'eau est produite par trois forages de types artésiens descendant jusqu'à 180 m sous le niveau du sol.

Station Kikwit ville :
Les forages de la station Kikwit ville sont équipés de pompes immergée permettant de pomper 95 et 125 m³/h. Soit un total de 200 m³ cube par heure. Il y a refoulement des eaux vers un réservoirs de 500 m³ sur le site même.
La chloration se fessait à l'époque grâce à une pompe doseuse qui est en panne depuis 2007. Aujourd'hui la chloration se fait de manière manuelle/ aléatoire. La quantité de chlore est versée dans le réservoirs au goute à goute grâce à un seau équipé d'un robinet.

Station Kikwit 3 :

Un forage artésiens . L'installation d'une pompe immergée est prévue mais pour le moment le refoulement des eaux se fait grâce à une pompe de surface. Chloration manuelle au niveau du réservoir.

Chloration du réseau :

La quantité de chlore utilisée chaque jours par la Regideso est insuffisante. Pas de chlore résiduel libre lors de nos test sur le réseau. Selon les jar test effectués lors de notre visite avec l'équipe de techniciens de la Regideso, il est nécessaire de chlorer l'eau à un dosage de 800 gr par 200m³.

Erosion du sol et protection du Réseau :

Il est à noter que les problèmes d'érosion du sol expose les canalisations aux risques de fuites. Les risque de contaminations du réseau est d'autant plus grand que le réseau n'est pas sous pression en permanence. La Regideso, doit souvent intervenir pour la réparation de celle ci.

Lors de nos visites plusieurs fuites ont été constatées sur le réseau et signalées à la Regideso, notamment dans la commune de Lukolela. La ligne passent entre les aires de santé de Lukolela et Maternité plateau devrait faire l'objet d'une révision.

D'autres part plusieurs points de distribution d'eau consistent uniquement en une sortie d'un tuyau du sol sur lequel est branché un tuyau flexible pour la distribution. Ce tuyau pouvant trainer au niveau du sol et compromet ainsi l'hygiène au point de distribution. Des bornes fontaines permettent d'assurer une bonne hygiène de distribution.

Autres :

- ⇨ Depuis 2002 la plupart des raccordements privés ont été supprimés et remplacés par des bornes fontaines publiques.
- ⇨ Le prix de l'eau a augmenté fortement ces dernières années.
- ⇨ Le projet PAIDECO de la CTB a financé la construction de 47 bornes fontaines après 2006.
- ⇨ Réhabilitation du réseau en cours
- ⇨ La construction d'un troisième site de production d'eau est prévue et devrait être financée par la coopération Japonaise.
- ⇨ Du gazoil est fourni par la Coopération Financière Allemande.

- Pas de CRL aux robinets au moment de nos tests. Sauf après augmentation du taux de chlore testée avec la Regideso lors de notre visite.
- Couverture limitée par un nombre de bornes fontaine publique insuffisant
- La population utilise la Regideso comme eau de boisson quand elle y a accès et quand elle a les moyens financiers.
- Coupures du réseau fréquentes, en générales plusieurs heures par jours.
- Présence de fuites sur le réseau (risque de contamination lorsque le réseau n'est pas sous pression)
- Prix de l'eau : 4000fc par m^3 (100 fc pour le bidon de 25 litres) (25 frc le bidon de 25 litres en 2006)
- Le réseau pourrait être une source de contamination contribuant à l'expansion de l'épidémie
- Des vérifications de la qualité bactérienne devrait être faite

Propositions :

En urgence et dans le cadre de l'intervention MSF FT Kikwit :
Une augmentation du taux de chlore sur le réseaux de manière à obtenir un résiduel de chlore au bornes fontaines pourrait prévenir d'une contamination de l'eau entre le point de distribution et sa consommation.
Nous proposons de faire une donation de 250 kg de chlore à la Regideso de Kikwit ainsi que de 3 pool testeur. Assurer un suivi de la chloration manuelle du réseau. (4 semaines)

Dans le long terme :

Chloration : Réparer la pompe doseuse et/ou la remplacer.

A prévoir : Renforcer la réfection des conduites existantes. Test d'étanchéité, réhabilitation de canalisation, chemisage de canalisation afin de prévenir les risques de contamination du réseau.
Lorsque il n'est pas possible d'enterrer à plus de 30 cm le tuyau d'adduction, utilisation de tuyau en acier galvanisé/ou la protection du tuyau en plastique par un remblai en béton est conseillé.

Réhabilitation des bornes fontaines et construction de nouvelles bornes fontaines. (hygiène au point de distribution).

Sources :
La population utilise également comme seconde source d'eau de nombreuses sources d'eau et résurgences dans le bas des pentes. Ces sources se situent au bord des ruisseaux et de la rivière Kwilu. Présence d'habitations en amont des sources. Présence de latrines en amont des sources, mais également pratique de la défécation à l'air libre.

- Assez nombreuses dans certains quartiers (4 à 5 par AdS ou elles sont les plus nombreuses)
- Utilisées comme sources d'eau potable là ou la Regideso est absente
- Débit généralement faible
- La plupart des sources ne sont pas aménagées et ne font pas l'objet de traitement ; elles sont utilisées pour l'eau de boisson
- Forte fréquentation de ces sources par la population
- Les sources sont souvent « gardées » et l'eau n'y est pas gratuite. Un bidon d'eau s'échange contre, un peu d'argent, une carotte de manioc, ou un peu de charbon.
- Lors des fortes pluies, eau de ruissellement peuvent contaminer de façon très importantes les sources.

Propositions :

En urgence et dans le cadre de l'intervention de MSF :
De manière à désinfecter une eau potentiellement contaminée par des germes d'origines fécales nous préconisons la mise en place de point chloration (chloration au seau) au niveau des sources des quartiers les plus touchés par la fièvre typhoïde. Possibilité d'utiliser pour les points de chloration de l'eau de javel disponible sur le marché local.
Une dizaine de point chloration au total. (4 semaines)

Dans le long terme :

Une meilleure protection des sources
=> Pas de latrines à moins de 30m des sources, mise en place d'un périmètre de protection de 30m autour des sources. Si possible clôturé.
=> Drainage des eaux de ruissellement autour des points d'eau

Hôpitaux de Kikwit Nord et Sud

Cfr fiche d'évaluation structure de santé watsan en annexe.

L'unique source d'eau au seins des hôpitaux de Kikwit nord et sud est la récupération d'eau de pluie. Ces hôpitaux sont équipés de réservoirs de grandes capacités. Cette eau est utilisée pour l'hygiène hospitalière. Les réservoirs sont encore en bon état, par contre les gouttières sont souvent endommagées. L'eau de boisson est récoltée par bidon à des bornes fontaine de la Regideso se situant à l'extérieur de l'hôpital.

Propositions :

En urgence et dans le cadre de l'intervention de MSF :
Nous proposons de réhabiliter/remplacer une partie des gouttières de manière à optimiser la présence de ces réservoirs d'eau.
Mise en place de First flush : entre chaque pluie , une couche de poussière se retrouve sur la toiture, pour améliorer la qualité de l'eau stockée nous proposons la mise en place de système de rinçage de la première pluie.
Nous proposons également de nettoyer et désinfecter les réservoirs des deux hôpitaux.
Mettre en place des points lavage de mains à la sortie de chaque latrine.

Dans le long terme :

Prévoir un raccordement du réseau d'eau au sein des hôpitaux.

Grand marché de Kikwit

- √ Pas d'eau distribuée au niveau du marché
  - ⇨ Pas d'eau disponible pour se laver les mains
  - ⇨ Un seul agent de propreté pour l'ensemble du marché
  - ⇨ Certains étalages sont au niveau du sol
  - ⇨ Présence d'un marché aménagé construit par la CTB mais pas encore en fonction.
  - ⇨ Présence d'un bloc de latrines construites par la CTB mais pas encore en service.

L'équipe de la CTB était en congés Noël lors de notre visite. Voir pour les rencontrer lors de l'intervention, pour avoir plus d'informations sur le travail qu'ils ont effectué pour un meilleur « environnement/hygiène » autour du marchés.

Promo santé

Lors de notre visite les activités de sensibilisation et de promo santé étaient quasi inexistantes. Faute de moyen.
Messages classiques d'hygiènes, lavage des mains, consommation de nourriture cuite,..

Propositions :

En urgence et dans le cadre de l'intervention de MSF :

En plus des messages classique pour l'hygiène.

Nous voudrions impliquer la promo santé dans les activités Watsan.
=> Acceptation de la population de la chloration (goût de l'eau) au niveau des bornes fontaines de la Regideso.
=> Utilisation de l'eau de javel pour désinfecter l'eau à domicile.

3. CONCLUSION ET RECOMMANDATIONS

- Probable que l'extension de l'épidémie soit liée à la consommation d'une eau contaminée, particulièrement au niveau des sources le long des ruisseaux en bas des pentes. Le début de l'épidémie correspondant au début de la saison des pluies, nous pensons que les eaux de ruissellement ont pu contaminer les sources via les latrines en amont.
- Quand le réseau n'est pas sous pression, les fuites sur le réseau d'eau de la Regideso représente un risque de contamination des eaux du réseaux.
- Le prix excessif de l'eau représente une contrainte pour l'accès à l'eau des populations. Augmentation du prix de l'eau de 400% en 5 ans.
- Le manque/l'absence de sanitaires propres sur les marchés, les écoles augmentent les risques de transmission de la maladie.

  - ⇨ La chloration du réseau peut prévenir des contaminations.
  - ⇨ Réparation de la pompe doseuse de la Regideso pour chloration
  - ⇨ Réhabilitation du réseau/ protection contre les fuites/éviter les coupures sur le réseau.
  - ⇨ La mise en place de points chloration au niveau de points d'eau dans les airs de santé les plus affectés
  - ⇨ Financement de projets de récupération d'eau de pluie dans les lieux publiques (écoles, pour améliorer l'hygiène (eau pour lavages des mains, nettoyage des latrines..)
