# Supplementary material for: Typhoid fever outbreak in the Democratic Republic of Congo: Case control and ecological study
Source: PLoS Negl Trop Dis. 2018 Oct 3;12(10):e0006795. doi: 10.1371/journal.pntd.0006795 (PMC6188896; doi:10.1371/journal.pntd.0006795)
Supplement: S1 Protocol — (DOC) [file pntd.0006795.s003.doc]

# Operational Research Study

## Title

**Typhoid Fever outbreak investigation. A case study to determine high transmission zones, Kikwit, DRC**

## Country

Democratic Republic of Congo

## Site

Kikwit

## Investigators and Institutions (alphabetic order)- to be confirmed

Anja De Weggheleire, Operational Department, Medecins sans Frontieres, Operational Center Brussels, Brussels, Belgium Medecins Sans Frontieres

Frederic Patigny; Medical Department, Medecins sans Frontieres, Operational Center Brussels, Brussels, Belgium Medecins Sans Frontieres

Mikael Mangold; Chalmers, Technical University, Gothenburg, Sweden

Paul R. Hunter; School of Medicine, Health Policy and Practice, University of East Anglia, Norwich, United Kingdom.

Peter Maes; Medical Department, Medecins sans Frontieres, Operational Center Brussels, Brussels, Belgium Medecins Sans Frontieres (P.I.)

Rafael Van Den Bergh; Medical department (Operational Research), Operational Center Brussels, MSF-Luxembourg, Luxembourg

Vincent Lambert; Operational Department, Medecins Sans Frontieres, Operational Center Brussels, Brussels, Belgium Medecins Sans Frontieres

To be identified: a non-MSF and MSF national co-author would be advisable

**Version Protocol:**

September 18, 2012 (substantially)

15 August 2018 (slight reformatting, grammar errors corrected, two authors’ margin comments were removed and any questions resolved).

**1. Introduction**

Typhoid Fever (TF) is a disease caused by the bacterium *Salmonella enterica* sv Typhi, which is contracted via faeco-oral transmission^1^. An inoculum as small as 100,000 organisms causes infection in more than 50% of healthy volunteers^2^. Humans are the only natural host and reservoir, and are infected through ingestion of faecally contaminated food or water. S. typhi may also be found in urine and vomitus of cases, and shellfish grown in sewage-contaminated water are potential vehicles, as are vegetables. Flies can mechanically transfer the organism to food, where the bacteria then multiply to achieve an infective dose. The highest incidence occurs where water supplies serving a large population are faecally contaminated. The incubation period is usually 8–14 days, but may range from 3 days up to 2 months. Some 2–5% of infected people become chronic carriers, who harbor *Salmonella* Typhi in the gall bladder. Chronic carriers are greatly involved in the spread of the disease^3,4^.

Clinical diagnosis of TF is challenging. In the absence of laboratory confirmation, any case of fever of at least 38 °C for 3 or more days is considered suspect if the epidemiological context is suggestive. Depending on the clinical setting and quality of available medical care, some 5– 10% of typhoid patients may develop serious complications, the most frequent being intestinal haemorrhage or peritonitis due to intestinal perforation.

An outbreak of TF has been recorded in Kikwit, Bandundu Province, in the Democratic Republic of Congo (DRC) in 2006 and 2011. Kikwit is the largest city of [Bandundu Province](http://en.wikipedia.org/wiki/Bandundu_(province)), lying on the [Kwilu River](http://en.wikipedia.org/wiki/Kwilu_River) in the southwestern part of the [Democratic Republic of Congo](http://en.wikipedia.org/wiki/Democratic_Republic_of_Congo). The population is approximately 350,000. Between September 4^th^ 2006 and January 7^th^ 2007, a total of 1802 cases were reported, mostly not confirmed by laboratory diagnosis. In total 973 simple TF cases and 77 cases with peritonitis and intestinal perforation have been registered. Approximately 25 patients died (fatality rate 1,3 %).

Figure 1 shows the epidemic curve for the Typhoid Fever outbreak from 4^th^ September 2006 until 7^th^ January 2007.

Since this reported outbreak in 2006, TF has been endemic in Kikwit, with limited cases reported in 2009, 2010 and the three first trimesters of 2011.

In week 46 of 2011, or five years after the first recorded outbreak, a second outbreak of TF occurred in Kikwit. Between November 14^th^ and December 25^th^ 2011, a total of 1438 cases were reported, of which 13 (1%) were confirmed by laboratory diagnosis: 6 were positive for *Salmonella* Typhi on 16 hemocultures and 7 were positive for *Salmonella* Typhi on 13 coprocultures. A total of 18 samples were rejected for analysis as not transported correctly. In total, 1367 simple TF cases and 71 cases with peritonitis and intestinal perforation were registered and approximately 22 patients have died (fatality rate 1,53 %).

Figure 3 shows the epidemic curve for the Typhoid Fever outbreak from November 14^th^ until 25^th^ December 2011.

Figures from the outbreak in 2006 and 2011 show an increase in cases of the disease at the beginning of the rainy season. Apart from this cyclic nature it can also be observed that in 2006 and 2011, the same geographic zones were systematically touched most; in these zones, a population of about 60,000 out of the 350,000 inhabitants of Kikwit are living. This information suggests that the TF outbreaks might be associated with a structural problem within a potential high TF risk zone in Kikwit.


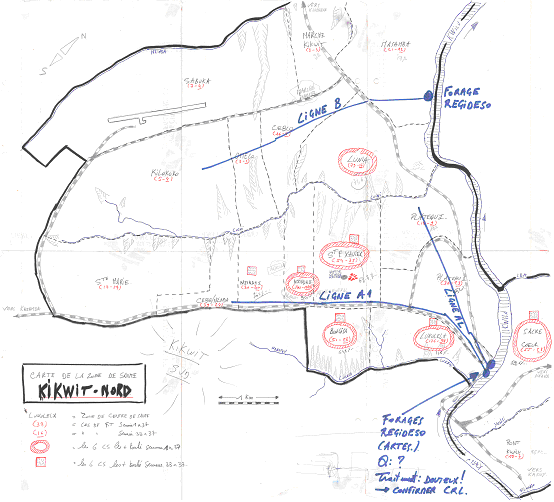


Figure 4: map of Kikwit Nord with hotspots of transmissions in 2006 and 2011 outbreaks

In Kikwit access to safe water is a challenge, and water source contamination is one of the most cited risk factors in a TF outbreak ^5^. During the rainy season, where there is either no sewage disposal or working sewer system, runoff of rains follows the slopes down to the Lukemi River and contaminates open water sources used by the population of “Kikwit ville basse”, the lower lying part of town, with excreta. Water produced at the borehole of Regisdeso at “Kikwit ville basse”, and sold to those who can afford it, might also be contaminated. Chlorine is not adequately applied and as the water pressure in the outdated and leaking network is not constant, the contaminated surface water will be sucked into the water pipes network the moment the pressure drops.

The central market, in proximity of this most affected “Kikwit ville basse” with its catastrophic hygienic circumstances and the lack of knowledge in the population about the transmission process of TF, probably also compounds the propagation of the pathogen in the population.

In this study we aim to address the following research question: can a detailed mapping of the cases of previous outbreaks provide a clearer understanding of the origin of the patients and determine zones of high TF transmission within Kikwit.

**2. Aim and Objectives:**

Through a detailed mapping of the cases of previous TF outbreaks, provide a clearer understanding of the origin of the patients and determine within Kikwit those zones of high TF transmission, where targeted Water, Hygiene and Sanitation activities would have the potential to prevent further outbreaks of TF in Kikwit

The three specific objectives of this analysis are:

2.1. Outbreak investigation: To describe the previous 2006 and 2011 outbreaks of Typhoid Fever in terms of person, place and time (age, sex, and geographic origin).

2.2. Hot spots of transmission: To verify on zones with high and systematic TF transmission during the 2006 and 2011 outbreaks in order to verify the existence of hot spots of TB transmission within the 33 “aires de santé” in Kikwit.

2.3. Environmental risk factors: To analyze the potential association between environmental risk factors (e.g. topographic elevation, proximity to river) and the clustering of TF cases amongst seven (approximately 60,000 inhabitants) out of the thirty-three (approximately 350,000 inhabitants) “aires de santé” of Kikwit during the 2006 and 2011 outbreak.

**3. Methods**

3.1. Study Design:

A descriptive study using routine project management and ecological data.

3.2. Setting:

Kikwit is the largest city of [Bandundu Province](http://en.wikipedia.org/wiki/Bandundu_(province)), lying on the [Kwilu River](http://en.wikipedia.org/wiki/Kwilu_River) in the southwestern part of the [Democratic Republic of Congo](http://en.wikipedia.org/wiki/Democratic_Republic_of_Congo). The population is approximately 350,000. The situation in RDC is dramatic in terms of health provision, with a weak health system, extremely poor accessibility of the existing health system and regular emergency levels of mortality figures, even in stable districts. These negative indicators are the result of, amongst others, decades of civil war and unrest, poor infrastructure and challenging geographical features, and poor management of the existing resources.

The sanitary district of Kikwit lies about 500 kilometers east of the capital, Kinshasa. It has a supervisee of 330 km² and a population density of approximately 1282 habitants per km². It is composed of 2 “zones de santé”:

- ZS de Kikwit Nord (161,046 habitants), 18 “aires de santé”, comprising 2 communes: Lukolela et Nzinda.
- ZS de Kikwit Sud (176,300 habitants), 15 “aires de santé”, comprising 2 communes: Lukeni et Kazamba

In Kikwit the environment is mountainous with in the extreme north the river Kwilu. Between the different mountains exist small rivers and springs. Availability of potable water is challenging especially in “Kikwit ville basse” the lower lying part of Kikwit town. Piped water is too expensive for most of the population and its quality is compromised by inappropriate chlorination, and a leaking distribution network that allows aspiration of surface water when the pumps stop turning and the water pressure drops within the network. The price of the water also compromises its accessibility considerably. As a coping mechanism the population uses open, unprotected springs to collect drinking water. Up to four or five springs can be identified per “aire de sante” and they well up at the bottom of the slopes in this mountainous environment. These sources are often situated close to small tributary rivers of the main river Kwilu. Only about 50 % of people in Kikwit own a latrine and wild defecation is rampant, also uphill of springs, leading to a high risk environment for TF transmission. The central market of Kikwit also has an important potential to maintain TF transmission, with an absence of water and hand washing points on the market itself. Only one individual is responsible for cleanness in the market and most goods are presented on the ground instead of on tables. No latrines are available on the market.

3.3. Site and study participants:

MSF is supporting Kikwit Nord General Hospital in order to offer free and quality treatment to simple TF patients as well as to those suffering from complications or perforation. Simple cases are given outpatient care and antibiotics, while complicated cases are hospitalized and perforated cases receive surgical treatment. MSF also provides medicines to the hospital, gives training to the personnel of the hospital and health centers around the city, and ensures that an adequate case definition and treatment protocol are implemented. Community workers are raising awareness among the population about basic hygiene practices, in order to halt this serious epidemic of TF, the so-called "dirty-hands disease”. These interventions are part of an emergency response, despite the fact that the problem is systemic and has repeated itself each 5 years in Kikwit.

All cases of the 2006 and 2011 TF outbreak will be included in the study. Environmental indicators will be provided across the 33 “aires the santé” and sample size calculations are thus not indicated.

3.4. Data variables

The data variables to be collected for this study are shown in relation to the objectives and are as follows:

3.4.1. Objective 1: data on incidence by age, sex and “aire de sante”, proportion of moderate and severe cases and weekly number of cases and the weekly incidence rates. Case definitions, provided jointly by the Ministry of Health and the World Health Organization, were: **Suspected case**: any person with gradual onset of steadily increasing and then persistently high fever, malaise, headache, sore throat, cough, and, sometimes, abdominal pain and constipation or diarrhea. **Confirmed case**: A suspected case confirmed by isolation of *Salmonella* Typhi from blood, bone marrow, duodenal fluid or stool. For the confirmation of TF, isolation of *Salmonella* Typhi is required. Blood culture is the mainstay of the diagnosis. In endemic areas stool isolation alone is insufficient for diagnosis if the clinical picture is not complete. Coproculture, antibiogram is confirmatory for carrier detection.

3.4.2. Objective 2: data on cases reported per 10,000 inhabitants in all the 33 “aires de santé” during the 2006 and 2011 TF outbreaks in Kikwit.

3.4.3. Objective 3: data on topographic elevation, proximity to river, population density and socio-economic factors (average income, education level) of the thirty-three (approximately 350,000 inhabitants) “aires de santé” of Kikwit during the 2006 and 2011 outbreak.

3.5. Sources of data

Sources of data are as follows: official data from the Ministry of Health registers (line list) will be used to describe the previous epidemics in terms of person, time and place. This line list has been created by the MOH in order to register every suspected case of TF, and contains several items of interest such as name, address, sex, age, date of onset, date of admission, symptoms at admission, status, results of laboratory analysis and outcome.

Population data (denominators) used for rate estimations will be obtained from the census bureau in Kikwit.

Complementary sources of information will be:

- interviews with the health authorities of the concerned area.
- environmental evaluation of the affected area.
- visit to the reference hospital.
- visit to the reference laboratory.

3.6 Analysis and statistics

Data will be double entered from the line lists, sitreps, interviews and technical reports into an EpiData database (EpiData Association, Odense, Denmark).

To describe the 2006 and 2011 TF outbreak, attack rates by age, sex and “aire de santé” will be calculated, as well as the proportions of moderate and severe cases; weekly numbers of cases, weekly incidence rates and case fatality ratios.

A comparison will be made of the cases reported per 10,000 inhabitants between the 33 “aires de sante” and during the two time periods using the chi-squared test for categorical variables. Levels of significance will be set at 5% (p < 0.05).

A multivariate analysis will be performed to examine the dependency of the attack rate per “aire de santé” (outcome) with several exposure values such as topographic elevation, proximity to river, population density and socio-economic factors (average income, education level) of the thirty-three “aires de santé” of Kikwit during the 2006 and 2011 outbreaks.

3.7 Ethics issues:

The study meets the following criteria for exemption from ERB review:

3.7.1. Informed consent: Only retrospective, routine data will be used in this study and will be presented as aggregate data, and no direct contact with individuals is required; informed consent is therefore not required.

3.7.2. Data confidentiality: All data and related forms will be held in strict confidence. No names of patients are recorded in the registers, and investigators will not be informed of the identity of any patients. No names or identifying information will be used in any publication or presentation and confidentiality of the patients can be assured.

3.7.3. Harm to study participants: The study will use existing, retrospective routine programme data and will therefore not influence current care. Risk for individual patients is thus minimal.

3.7.4. Benefits for the community: Spatial analysis is a simple but powerful tool, which might be able to identify potential hot spots of transmission and thus help focus water, sanitation and health education interventions, which might limit the spread of a potential future outbreak.

3.7.5. Community participation in project development and progress: In a later stage of the study, stakeholder participation is important to assure the correct utilization of the targeted Water and Sanitation infrastructures to promote hygiene and health of the target population.

3.7.6. Dissemination of results, including to participants and local communities: The results of the study will be made known to stakeholders in the affected study area. In order to disseminate the information gathered, attempts will be made to present the study at national and international conferences and publish the study in regional or international peer reviewed journals. Communication will be focused in particular towards other partners involved in TF prevention. The German Technical Cooperation expressed its interest in this approach as the result of this study might assist in obtaining the funds required to improve the water distribution infrastructure and help this interested implementing partner to orientate their wash activities in Kikwit, instead of going blind.

3.7.7. Implication for policy and practice: The study is expected to have policy and programme implications for humanitarian and UN organizations as well as for government of countries facing recurrent TF outbreaks.

3.7.8. Collaborative partners: The data were collected in collaboration with the MOH. National co-investigators will be involved in the study. Representatives of collaborative institutions will be represented on the study manuscript and any eventual publication.

## References

1. WHO, Guidelines for the Management of Typhoid Fever, 2011.

2. Levine, MM, et al.,. Host-Salmonella interaction: human trials. *Microbes Infect*. Nov-Dec 2001;3(14-15):1271-9.

3. Parry, C. et al., Typhoid Fever . N Engl J Med, 2002, Vol. 347, No. 22 November 28, 2002

4. Bhan M.K. et al. Typhoid and paraTyphoid Fever, the lancet.com Vol 366 August 27, 2005

5. Kariuki, S., Typhoid Fever in sub-Saharan Africa: Challenges of diagnosis and management of infections. J Infect Developing Countries 2008; 2(6):443-447.
