# Supplementary material for: Typhoid fever outbreak in the Democratic Republic of Congo: Case control and ecological study
Source: PLoS Negl Trop Dis. 2018 Oct 3;12(10):e0006795. doi: 10.1371/journal.pntd.0006795 (PMC6188896; doi:10.1371/journal.pntd.0006795)
Supplement: S2 Protocol — (DOC) [file pntd.0006795.s004.doc]

# Operational Research Study

## Title

**Typhoid Fever outbreak investigation. A case study to determine water and sanitation-related risk factors for Typhoid Fever transmission during the 2011 outbreak in Kikwit, DRC**

## Country

Democratic Republic of Congo

## Site

Kikwit

## Investigators and Institutions (alphabetic order)- to be confirmed

Anja De Weggheleire, Operational Department, Medecins sans Frontieres, Operational Center Brussels, Brussels, Belgium Medecins Sans Frontieres

Frederic Patigny; Medical Department, Medecins sans Frontieres, Operational Center Brussels, Brussels, Belgium Medecins Sans Frontieres

Mikael Mangold; Chalmers, Technical University, Gothenburg, Sweden

Paul R. Hunter; School of Medicine, Health Policy and Practice, University of East Anglia, Norwich, United Kingdom.

Peter Maes; Medical Department, Medecins sans Frontieres, Operational Center Brussels, Brussels, Belgium Medecins Sans Frontieres (P.I.)

Rafael Van Den Bergh; Medical department (Operational Research), Operational Center Brussels, MSF-Luxembourg, Luxembourg

Vincent Lambert; Operational Department, Medecins Sans Frontieres, Operational Center Brussels, Brussels, Belgium Medecins Sans Frontieres

To be identified: a non-MSF and MSF national co-author would be advisable

**Version Protocol:**

September 18, 2012 (substantially)

15 August 2018 (slight reformatting, grammar errors corrected, one author margin comments was removed with its questions resolved).

**1. Introduction**

Typhoid Fever (TF) is a disease caused by the bacterium *Salmonella enterica* sv Typhi, which is contracted via faeco-oral transmission^1^. An inoculum as small as 100,000 organisms causes infection in more than 50% of healthy volunteers^2^. Humans are the only natural host and reservoir, and are infected through ingestion of faecally contaminated food or water. S. typhi may also be found in urine and vomitus of cases, and shellfish grown in sewage-contaminated water are potential vehicles, as are vegetables. Flies can mechanically transfer the organism to food, where the bacteria then multiply to achieve an infective dose. The highest incidence occurs where water supplies serving a large population are faecally contaminated. The incubation period is usually 8–14 days, but may range from 3 days up to 2 months. Some 2–5% of infected people become chronic carriers, who harbor *Salmonella* Typhi in the gall bladder. Chronic carriers are greatly involved in the spread of the disease^3,4^.

Clinical diagnosis of TF is challenging. In the absence of laboratory confirmation, any case of fever of at least 38 °C for 3 or more days is considered suspect if the epidemiological context is suggestive. Depending on the clinical setting and quality of available medical care, some 5– 10% of typhoid patients may develop serious complications, the most frequent being intestinal haemorrhage or peritonitis due to intestinal perforation.

An outbreak of TF has been recorded in Kikwit, Bandundu Province, in the Democratic Republic of Congo (DRC) in 2006 and 2011. Kikwit is the largest city of [Bandundu Province](http://en.wikipedia.org/wiki/Bandundu_(province)), lying on the [Kwilu River](http://en.wikipedia.org/wiki/Kwilu_River) in the southwestern part of the [Democratic Republic of Congo](http://en.wikipedia.org/wiki/Democratic_Republic_of_Congo). The population is approximately 350,000. Since the 2006 reported outbreak, TF has been endemic in Kikwit, with limited cases reported in 2009, 2010 and the 3 first trimestres of 2011.

Figure 1 shows the epidemic curve for Typhoid Fever cases from January 1, 2009 to December 31, 2011.

In week 46 of 2011, or five years after the first recorded outbreak, a second outbreak of TF occurred in Kikwit. Between November 14^th^ and December 25^th^ 2011, a total of 1438 cases were reported, of which 13 (1%) were confirmed by laboratory diagnosis: 6 were positive for *Salmonella* Typhi on 16 hemocultures and 7 were positive for *Salmonella* Typhi on 13 coprocultures. A total of 18 samples were rejected for analysis as not transported correctly. In total, 1367 simple TF cases and 71 cases with peritonitis and intestinal perforation were registered and approximately 22 patients have died (fatality rate 1,53 %).

Figure 2 shows the epidemic curve for the Typhoid Fever outbreak from November 14^th^ until 25^th^ December 2011.

Figures from the outbreak in 2006 and 2011 show an increase in cases of the disease at the beginning of the rainy season. Apart from this cyclic nature it can also be observed that in 2006 and 2011, the same geographic zones were systematically touched most; in these zones, a population of about 60,000 out of the 350,000 inhabitants of Kikwit are living. This information suggests that the TF outbreaks might be associated with a structural problem within a potential high TF risk zone in Kikwit. This hypothesis, that 7 out of the 33 “aires de santé” represent a hotspot of TF transmission within Kikwit, is currently the topic of a parallel focused investigation.


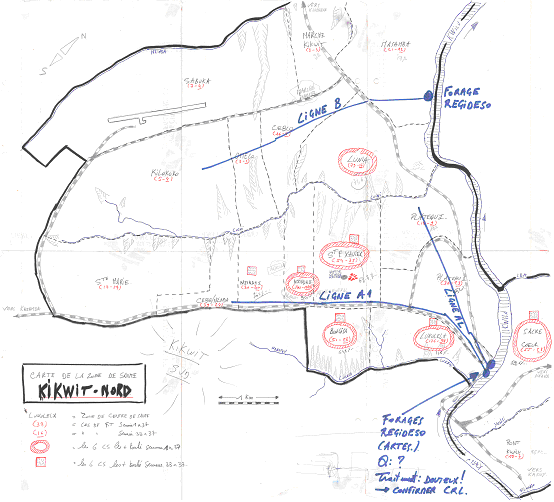


Figure 3: map of Kikwit Nord with hotspots of transmissions in 2006 and 2011 outbreaks

In Kikwit access to safe water is a challenge, and water source contamination is one of the most cited risk factors in a TF outbreak^5^. During the rainy season, where there is either no sewage disposal or working sewer system, runoff of rains follows the slopes down to the Lukemi River and contaminates open water sources used by the population of “Kikwit ville basse”, the lower lying part of town, with excreta. Even water produced at the borehole of Regisdeso at “Kikwit ville basse”, and sold to those who can afford it, might be contaminated. Chlorine is not adequately applied and as the water pressure in the outdated and leaking network is not constant, the contaminated surface water is sucked into the pipes the moment the pressure drops.

The central market, in proximity of this most affected “Kikwit ville basse” with its catastrophic hygienic circumstances, and the lack of knowledge in the population about the transmission process of TF, probably also compounds the propagation of the pathogen in the population.

In this study, we aim to investigate risk factors of TF transmission within a limited zone of Kikwit where TF transmission is particularly high in order to add to the growing evidence base on TF epidemiology.

**2. Aim and Objectives:**

Identify risk factors of TF transmission within a limited zone of Kikwit, where TF transmission was particularly high, in order to provide recommendations for future interventions against TF outbreaks in Kikwit and similar contexts.

**3. Methods**

3.1. Study Design:

A observational case-control study with individual matching for age and sex.

3.2. Setting:

Kikwit is the largest city of [Bandundu Province](http://en.wikipedia.org/wiki/Bandundu_(province)), lying on the [Kwilu River](http://en.wikipedia.org/wiki/Kwilu_River) in the southwestern part of the [DRC](http://en.wikipedia.org/wiki/Democratic_Republic_of_Congo). The population is approximately 350,000. The situation in DRC is dramatic in terms of health provision, with a weak health system, extremely poor accessibility of the existing health system and regular emergency levels of mortality figures, even in stable districts. These negative indicators are the result of, amongst others, decades of civil war and unrest, poor infrastructure and challenging geographical features, and poor management of the existing resources.

The sanitary district of Kikwit lies about 500 kilometers east of the capital, Kinshasa. It has a supervisee of 330 km² and a population density of approximately 1282 habitants per km². It is composed of 2 “zones de santé”:

- ZS de Kikwit Nord (161,046 habitants), 18 “aires de santé”, comprising 2 communes: Lukolela et Nzinda.
- ZS de Kikwit Sud (176,300 habitants), 15 “aires de santé”, comprising 2 communes: Lukeni et Kazamba

In Kikwit, the environment is mountainous with in the extreme north the river Kwilu. Between the different mountains exist small rivers and springs. Availability of potable water is challenging, especially in “Kikwit ville basse”, the lower lying part of Kikwit town. Piped water is too expensive for most of the population and its quality is compromised by inappropriate chlorination, and a leaking distribution network that allows aspiration of surface water when the pumps stop turning and the water pressure drops within the network. The price of the water also compromises its accessibility considerably. As a coping mechanism, the population uses open, unprotected springs to collect drinking water. Up to four or five springs can be identified per “aire de sante” and they well up at the bottom of the slopes in this mountainous environment. These sources are often situated close to small tributary rivers of the main river Kwilu. Only about 50 % of people in Kikwit own a latrine and wild defecation is rampant, also uphill of springs, leading to a high-risk environment for TF transmission. The central market of Kikwit also has an important potential to maintain TF transmission, with an absence of water and hand washing points on the market itself. Only one individual is responsible for cleanness in the market and most goods are presented on the ground instead of on tables. No latrines are available on the market.

3.3. Study site:

MSF is supporting Kikwit Nord General Hospital in order to offer free and quality treatment to simple TF patients as well as to those suffering from complications or perforation. Simple cases are given outpatient care and antibiotics, while complicated cases are hospitalized and perforated cases receive surgical treatment. MSF also provides medicines to the hospital, gives training to the personnel of the hospital and health centers around the city, and ensures that an adequate case definition and treatment protocol are implemented. Community workers are raising awareness among the population about basic hygiene practices, in order to halt this serious epidemic of TF, the so-called "dirty-hands disease”. These interventions are part of an emergency response, despite the fact that the problem is systemic and has repeated itself each 5 years in Kikwit.

## 3.4. Study population

The study population consisted of TF cases randomly selected from the line list of cases who are living within 7 out of the 33 “aires de santé”, believed to be hotspots of TF transmission within Kikwit.

The case definition during the outbreak was provided jointly by the Ministry of Health and the World Health Organization. A **Suspected case** is any person with gradual onset of steadily increasing and then persistently high fever, malaise, headache, sore throat, cough, and, sometimes, abdominal pain and constipation or diarrhea. A **Confirmed case** is a suspected case confirmed by isolation of *Salmonella* Typhi from blood, bone marrow, duodenal fluid or stool. For the confirmation of TF, isolation of *Salmonella* Typhi is required. Blood culture is the mainstay of the diagnosis. In endemic areas, stool isolation alone is insufficient for diagnosis if the clinical picture is not complete.

Two controls of the same sex, age class (10 to 14, 15 to 19, 20 to 29, 30 to 39, 40 to 49 and more than 50) and “aire de santé” will be selected per case. Selection criteria for controls are not having been suspected of TF during the study period and having the possibility to present themselves at the health structure during the study period if required.

## 3.5. Sample size

Two controls will be selected for each case. In order to identify an Odds Ratio of at least 1.5 with a power of 80% for risk factors present in 30% of the control population, a sample size of 320 cases and 640 controls was calculated.

## 3.6. Sampling procedure

A representative sample of cases will be selected by random sampling, using random numbers generated in MS Excel, from a line list of cases who are living within the 7 “aires de santé” believed to be hotspots of TF transmission within Kikwit. Controls will be selected in two phases.

In the first phase, a geo-referenced polygon around each of the the 7 “aires de sante” will be generated from images obtained from Google Earth. Then with the help of the software package R, a total of 1500 random points will be generated within this polygon and the geographic coordinates of each point will be registered. Again, with the help of Google Earth images, all the points not falling on the roof of a house will be eliminated. Finally, 640 houses will be selected at random from the remaining points and allocated to cases: the first and the second point will be allocated to case 1, the third and fourth to case 2, and so on. As such, each case of the study population will have 2 random points allocated that each correspond to a house within the polygon.

In the second phase, with the help of a GPS, the corresponding houses will be visited by field teams. Within the household, a control will be recruited, matching the corresponding case in sex and age, and without suspicion of TF during the study period. If within this household no one matches the case or no one corresponds to the control criteria, then the field team will survey the next occupied building to the right. A household is defined as a group of people sleeping in the house and sharing the meals regularly during the study period.

## 3.7. Study period

## This case control survey will be carried out in Kikwit between November 2012 and February 2013 and is related to the TF outbreak that struck Kikwit between November 14^th^ and December 25^th^ 2011.

## 3.8. Study procedures

A semi-structured questionnaire was developed to gather information on the presence or absence of TF morbidity in conjunction with various factors (basic socio-demographics; water source/s, storage capacity, and point-of-use treatment; hygiene and sanitation practices; food and drink consumption; and health seeking behavior). The number of questions was limited to be amenable. The survey questionnaire is provided as Annex I. Information was collected both at the level of individual respondent and of each household as a whole. On consultation with national staff, the minimum age for respondents was set at 10 years.

The questionnaire was translated from English to French, back-translated by a second translator for verification, and piloted in an area of Kikwit not included in the selected sample. The survey team will comprise of residents of Kikwit (both men and women), coordinated by supervisors. The supervisors will control the quality of completed forms and data entry, and additional random checks will be carried out by the survey coordinator.

## Households will be selected as described above and interviews will be conducted. Interviewers will request to be shown any household water storage receptacles and latrines present at the end of each interview. The locations of principal water sources for each household will be identified using information collected in the questionnaire, and their positions will be recorded using hand-held Global Positioning System (GPS) devices. Locations will be cross-referenced, where possible, against information available from other actors for verification purposes.

3.9. Sources of data

Sources of data are as follows: official data from the Ministry of Health registers (line list) will be used to describe the previous epidemics in terms of person, time and place. This line list has been created by the MOH in order to register every suspected case of TF, and contains several items of interest such as name, address, sex, age, date of onset, date of admission, symptoms at admission, status, results of laboratory analysis and outcome.

Population data (denominators) used for rate estimations will be obtained from the census bureau in Kikwit.

Complementary sources of information will be:

- interviews with the health authorities of the concerned area.
- environmental evaluations in the most affected area’s.
- visit to the reference hospital.
- visit to the reference laboratory.
- chemical and biological water tests.

## 3.10. Chemical and biological water tests

## No major improvements have been made to the water distribution infrastructure since November 2011, and water quality testing can thus still be performed, as results are likely to reflect the conditions of 2011. Two tests will be used to evaluate the quality of water being consumed: a test to measure the free residual chlorine and a test to evaluate the presence of coliform bacteria. The chlorine test will be performed on water samples collected from the household water reservoir and when possible from the water source used by the household. This will be done using a spectrometer HANNA HI 701 Checker © HC² (HANNA ©Instruments, USA). The level of 0,2 mg/l will be considered as sufficient to consider the water as potable. The test of presence of coliforms will be done only on a water sample collected from the household water reservoir with the help of the test AquaCHROM ^TM^ (CHROMagar TM, France).

## 3.11. Main outcome measures

The main outcome measures in this study is TF incidence, both simple cases and cases with peritonitis and intestinal perforation.

## 3.12. Variables

For each respondent, all variables contained in the survey questionnaire will be collected. The study is centered on demographics and household composition, household water sourcing and storage, hygiene practices, and food/drink hygiene practices. The variables collected are listed in full in the questionnaire in Annex I. Additionally, variables pertaining to water quality will be collected per household (cf. §3.10.).

## 3.13. Data and statistical analysis

Questionnaire results will be encoded in full. Data will be processed and analysed (bivariate and multivariate analysis) using Epi-Info and STATA software packages. For all individual variables, results will be analyzed as three groups, respondents contracting simple, complicated or no TF during the 2011 outbreak.

4. Ethics issues:

The general medical and sanitation activities during the Kikwit Typhoid Fever outbreak response were part of the overall MSF emergency response and were in line with standard MSF conduct. General measures were in place to ensure confidentiality and support for all patients, as is standard MSF practice, and these measures will also be respected while conducting the survey. All care provided to the patients was free of charge, as standard for MSF. Data included in this analysis will not include patient identifiers. The nature and purpose of the survey will be explained to each household and verbal consent will be obtained from every individual prior to survey administration. Ethical approval will be sought from the Ethics Review Boards of MSF and the Ministry of Health of DRC in order to perform the analysis of the survey in full compliance with the ethical requirements within DRC and MSF.

## 4.1. Informed consent

The nature and purpose of the survey will be explained to each household. Participation is entirely voluntary and verbal consent will be obtained from every individual prior to survey administration. The survey is strictly verbal and will in no way caused any harm to its participants. All data will remain confidential and will be used for the sole purpose of this study. Personal identifiers will be removed from the final data set prior to analysis.

## 4.2. Community benefits

As the analysis of the survey will be performed retrospectively, there will be no direct benefits for the involved respondents. However, identification of putative risk factors and associations for cholera transmission in Kikwit will allow improvement of the TF intervention in Kikwit, and can as such lead to improved living conditions and increased protection against TF in the community.

## 4.3. Feedback & dissemination

Results of this study will be shared within the MSF program in DRC, and in case gaps or markedly strong points of the MSF intervention are identified, the relevant protocols on TF management and Water and Sanitation measures will be accordingly revised. Results will also be disseminated to the Ministry of Health, local partners (such as Oxfam and Unicef) and local and international policy makers involved in TF outbreak responses. We will aim to publish the study findings in a regional or international peer reviewed journal (open access) and to present the findings at relevant forums. Communication will be focused in particular towards other partners involved in TF prevention. The German Technical Cooperation expressed its interest in this approach as the result of this study might assist in obtaining the funds required to improve the water distribution infrastructure and help this interested implementing partner to orientate their wash activities in Kikwit, instead of going blind.

## 4.4. Implications for policy and practice

The findings of this study can be directly relevant to the Water and Sanitation guidelines for TF outbreak interventions and may lead to updating/fine-tuning of these guidelines. Additionally, the putative identification of novel risk factors for Typhoid Fever transmission can be used to guide the Water and Sanitation activities of other actors in the field.

## 4.5. Collaborative partnerships

The study will involve collaborating partners of the DRC Ministry of Health. Representatives of all collaborative institutions will be represented on the study manuscript and eventual publication.

# 4.6. Budget

The costs of conducting the survey and analysing the results will be carried by the operational cell 1 of MSF-Operational Centre Brussels. Funding for eventual publication in an open access journal will be provided by the MSF-Luxembourg Operational Research unit (LUXOR).

5. References

1. WHO, Guidelines for the Management of Typhoid Fever, 2011.

2. Levine, MM, et al.,. Host-Salmonella interaction: human trials. *Microbes Infect*. Nov-Dec 2001;3(14-15):1271-9.

3 Parry, C. et al., Typhoid Fever . N Engl J Med, 2002, Vol. 347, No. 22 November 28, 2002

4 Bhan M.K. et al. Typhoid and paraTyphoid Fever, the lancet.com Vol 366 August 27, 2005

5. Kariuki, S., Typhoid Fever in sub-Saharan Africa: Challenges of diagnosis and management of infections. J Infect Developing Countries 2008; 2(6):443-447.
