# Supplementary material for: Typhoid fever outbreak in the Democratic Republic of Congo: Case control and ecological study
Source: PLoS Negl Trop Dis. 2018 Oct 3;12(10):e0006795. doi: 10.1371/journal.pntd.0006795 (PMC6188896; doi:10.1371/journal.pntd.0006795)
Supplement: S1 Questionnaire — (DOC) [file pntd.0006795.s005.doc]

Moi ___________________________________________ (nom de l'enquêteur) déclare que la personne interrogée a expressément déclaré d’avoir reçu suffisamment d'informations sur les raisons pourquoi il/elle est invitée à participer à cette étude sur le risque de fièvre typhoïde. Il/elle comprend que la participation à cette étude est strictement volontaire et il/elle consente à sa participation.

Signature Date

| **Interview et observation des ménages**  **Enquête sur l’épidémie de fièvre typhoïde – Kikwit / RD Congo / 2013** | | | | | | | | | | | | | | | | | | | | | | | | | | |
| --- | --- | --- | --- | --- | --- | --- | --- | --- | --- | --- | --- | --- | --- | --- | --- | --- | --- | --- | --- | --- | --- | --- | --- | --- | --- | --- |
| MSF-OCB (Unité Eau Hygiène Assainissement) | | | | | | | | | | | | | | | | | | | | | | | | | | |
|  | | | | | | | | | | | | | | | | | | | | | | | | | | |
| **Q1** | | **Date de la visite :** | | | | | | | | | |  | | | |  | |  | | | |  | | |  | |
|  | |  | | | | |  | | | | |  | | | |  | |  | | | |  | | |  | |
| **Q2** | | **Numéro de la famille :** | | | | | | | | | |  | | | |  | |  | | | |  | | |  | |
|  | |  | | | | |  | | | | |  | | | |  | |  | | | |  | | |  | |
| **Q3** | | **Numéro de l’équipe :** | | | | | | | | | |  | | | |  | |  | | | |  | | |  | |
|  | |  | | | | |  | | | | |  | | | |  | |  | | | |  | | |  | |
| **Q4** | | **Coordonnées GPS** | | | | | | | | | | No : | | | |  | |  | | | |  | | |  | |
|  | |  | | | | |  | | | | |  | | | |  | |  | | | |  | | |  | |
| **Q5** | | **Topographie : décrire la topographie à l’échelle locale. L’habitation se situe :** | | | | | | | | | | | | | | | | | | | | | | | | |
|  | |  | | | | | O | | Sur une crête (même très locale) | | | | | | | | | | | | | | | | |  |
|  | |  | | | | | O | | Sur une pente | | | | | | | | | | | | | | | | |  |
|  | |  | | | | | O | | Dans un fond (même très local) | | | | | | | | | | | | | | | | |  |
|  | |  | | | | |  | | | | |  | | | |  | |  | | | |  | | |  | |
| **GÉNÉRALITÉS** | | | | | | | | | | | | | | | | | | | | | | | | | | |
|  | |  | | | | |  | | | | |  | | | |  | |  | | | |  | | |  | |
| **Q6** | | **Type d’habitation : (une seule réponse)** | | | | | | | | | | | | | | | |  | | | |  | | |  | |
|  | |  | | | | | O | | Murs de boue / non consolidés | | | | | | | | | | | | | | | | | 1 |
|  | |  | | | | | O | | Murs de boue / consolidés | | | | | | | | | | | | | | | | | 2 |
|  | |  | | | | | O | | Murs de briques / non consolidés | | | | | | | | | | | | | | | | | 3 |
|  | |  | | | | | O | | Murs de briques / consolidés | | | | | | | | | | | | | | | | | 4 |
|  | |  | | | | | O | | Habitation à plusieurs niveaux | | | | | | | | | | | | | | | | | 5 |
|  | |  | | | | | O | | Autre (préciser) : | | | | | | | | | | | | | | | | | 50 |
|  | |  | | | | | O | | Pas de réponse | | | | | | | | | | | | | | | | | 99 |
|  | |  | | | | |  | | | | |  | | | |  | |  | | | |  | | |  | |
| **Q7** | | **Combien de personnes votre ménage compte-t-il ?** | | | | | | | | | | | | | | | | | | | | | | | | |
|  | |  | | | | | Réponse : | | | | |  | | | |  | |  | | | |  | | |  | |
|  | |  | | | | |  | | | | |  | | | |  | |  | | | |  | | |  | |
| **Q8** | | **Quelle est la principale occupation du chef de ménage ? (une seule réponse)** | | | | | | | | | | | | | | | | | | | | | | | | |
|  | |  | | | | | O | | Travailleur journalier occasionnel (sans contrat) | | | | | | | | | | | | | | | | | 1 |
|  | |  | | | | | O | | Travailleur sous contrat | | | | | | | | | | | | | | | | | 2 |
|  | |  | | | | | O | | Propriétaire de sa propre entreprise | | | | | | | | | | | | | | | | | 3 |
|  | |  | | | | | O | | Agriculteur | | | | | | | | | | | | | | | | | 4 |
|  | |  | | | | | O | | Autre (préciser) : | | | | | | | | | | | | | | | | | 50 |
|  | |  | | | | | O | | Pas de réponse | | | | | | | | | | | | | | | | | 99 |
|  | |  | | | | |  | | | | |  | | | |  | |  | | | |  | | |  | |
| **Q9** | | **Savez-vous lire et écrire ? (La question est à poser au chef de ménage)** | | | | | | | | | | | | | | | | | | | | | | | | |
|  | |  | | | | | O | | Oui | | | | | | | | | | | | | | | | | 1 |
|  | |  | | | | | O | | Non | | | | | | | | | | | | | | | | | 2 |
|  | |  | | | | | O | | Pas de réponse | | | | | | | | | | | | | | | | | 99 |
|  | |  | | | | |  | | | | |  | | | |  | |  | | | |  | | |  | |
| **Q10** | | **Si vous êtes allé à l’école, quel niveau avez-vous atteint ? (La question est à poser au chef de ménage)** | | | | | | | | | | | | | | | | | | | | | | | | |
|  | |  | | | | | O | | Niveau primaire (terminé) | | | | | | | | | | | | | | | | | 1 |
|  | |  | | | | | O | | Niveau primaire (non terminé) | | | | | | | | | | | | | | | | | 2 |
|  | |  | | | | | O | | Niveau secondaire / Études professionnelles (terminé) | | | | | | | | | | | | | | | | | 3 |
|  | |  | | | | | O | | Niveau secondaire / Études professionnelles (non terminé) | | | | | | | | | | | | | | | | | 4 |
|  | |  | | | | | O | | Université (terminé) | | | | | | | | | | | | | | | | | 5 |
|  | |  | | | | | O | | Université (non terminé) | | | | | | | | | | | | | | | | | 6 |
|  | |  | | | | | O | | Pas de réponse | | | | | | | | | | | | | | | | | 99 |
|  | |  | | | | |  | | | | |  | | | |  | |  | | | |  | | |  | |
| **Q11** | | **Votre ménage dispose-t-il de l’un des éléments suivants ?** | | | | | | | | | | | | | | | | | | | | | | | | |
|  | |  | | | | |  | | | | | | | | | Oui  1 | | | Non  2 | | | | Pas de réponse  99 | | | |
|  |  |  |  |  |  |  |  |  |  |  |  |  |  |  |  |  |  |  |  |  |  |  |  |  |  |  |
|  | |  | | | | | Électricité en état de fonctionnement | | | | | | | | |  | | |  | | | |  | | | |
|  | |  | | | | | Radio en état de fonctionnement | | | | | | | | |  | | |  | | | |  | | | |
|  | |  | | | | | Télévision en état de fonctionnement | | | | | | | | |  | | |  | | | |  | | | |
|  | |  | | | | | Téléphone portable | | | | | | | | |  | | |  | | | |  | | | |
|  | |  | | | | |  | | | | |  | | | |  | |  | | | |  | | |  | |
| **Q12** | | **Si le questionnaire est adressé à une femme adulte, lui poser la question suivante :  Étiez-vous enceinte au moment de les dernières élections présidentielles?** | | | | | | | | | | | | | | | | | | | | | | | | |
|  | |  | | | | | O | | Oui | | | | | | | | | | | | | | | | | 1 |
|  | |  | | | | | O | | Non | | | | | | | | | | | | | | | | | 2 |
|  | |  | | | | | O | | Autre (préciser) | | | | | | | | | | | | | | | | | 50 |
|  | |  | | | | | O | | Pas de réponse | | | | | | | | | | | | | | | | | 99 |
|  | |  | | | | |  | | | | |  | | | |  | |  | | | |  | | |  | |
| **Q13** | | **Savez-vous comment éviter la fièvre typhoïde ?** | | | | | | | | | | | | | | | | | | | | | | | | |
| Si « Oui », passer à la question 14  Si « Non », passer directement à la question 16 | | | | | | | O | | Oui | | | | | | |  | |  | | | |  | | | | 1 |
|  |  |  |  |  |  |  | O | | Non | | | | | | |  | |  | | | |  | | | | 2 |
|  |  |  |  |  |  |  | O | | Autre (préciser) | | | | | | |  | |  | | | |  | | | | 50 |
|  |  |  |  |  |  |  | O | | Pas de réponse | | | | | | |  | |  | | | |  | | | | 99 |
|  | |  | | | | |  | | | | |  | | | |  | |  | | | |  | | |  | |
| **Q14** | | **Pouvez-vous énumérer les mesures préventives contre la fièvre typhoïde: (plusieurs réponses)** | | | | | | | | | | | | | | | | | | | | | | | | |
|  | |  | | | | | O | Traitement de l’eau | | | | | | | | | | | | | | | | | | 1 |
|  | |  | | | | | O | Lavage des mains | | | | | | | | | | | | | | | | | | 2 |
|  | |  | | | | | O | Utilisation des latrines | | | | | | | | | | | | | | | | | | 3 |
|  | |  | | | | | O | Consommation d’aliments chauds | | | | | | | | | | | | | | | | | | 4 |
|  | |  | | | | | O | Vaccination | | | | | | | | | | | | | | | | | | 5 |
|  | |  | | | | | O | Autre (préciser) | | | | | | | | | | | | | | | | | | 50 |
|  | |  | | | | | O | Pas de réponse | | | | | | | | | | | | | | | | | | 99 |
|  | |  | | | | |  | | | | |  | | | |  | |  | | | |  | | |  | |
| **Q15** | | **Avant les dernières élections présidentielles, saviez-vous comment éviter la fièvre typhoïde ?** | | | | | | | | | | | | | | | | | | | | | | | | |
|  | |  | | | | | O | Oui | | | | | | | |  | |  | | | |  | | | | 1 |
|  | |  | | | | | O | Non | | | | | | | |  | |  | | | |  | | | | 2 |
|  | |  | | | | | O | Autre (préciser) | | | | | | | |  | |  | | | |  | | | | 50 |
|  | |  | | | | | O | Pas de réponse | | | | | | | |  | |  | | | |  | | | | 99 |
|  | |  | | | | |  | | | | |  | | | |  | |  | | | |  | | |  | |
| **QUALITÉ, QUANTITÉ ET ACCESSIBILITÉ DE L’EAU** | | | | | | | | | | | | | | | | | | | | | | | | | | |
|  | |  | | | | | | | | | | | | | | | | | | | | | | | | |
| **Q16** | | **Quelle est votre principale source (utiliser souvent) d’eau (de boisson) ? (une seule réponse)** | | | | | | | | | | | | | | | | | | | | | | | | |
|  | |  | O | | L’eau du robinet de mon habitation | | | | | | | | | | | | | | | | | | | | | 1 |
|  | |  | O | | L’eau du robinet aux points de distribution communaux (REGIDESO) | | | | | | | | | | | | | | | | | | | | | 2 |
|  | |  | O | | Vendeurs dans la rue | | | | | | | | | | | | | | | | | | | | | 3 |
|  | |  | O | | Source protégée | | | | | | | | | | | | | | | | | | | | | 4 |
|  | |  | O | | Source non protégée | | | | | | | | | | | | | | | | | | | | | 5 |
|  | |  | O | | Puits équipé d’une pompe à main | | | | | | | | | | | | | | | | | | | | | 6 |
|  | |  | O | | Puits non équipé d’une pompe à main | | | | | | | | | | | | | | | | | | | | | 7 |
|  | |  | O | | Eau de surface – Préciser le nom du cours d’eau : | | | | | | | | | | | | | | | | | | | | | 8 |
|  | |  |  | |  | | | | | O | | | Kwilu | | | | | | | | | | | | | 8.1 |
|  | |  |  | |  | | | | | O | | | Lukemi | | | | | | | | | | | | | 8.2 |
|  | |  |  | |  | | | | | O | | | Luini | | | | | | | | | | | | | 8.3 |
|  | |  |  | |  | | | | | O | | | Iba | | | | | | | | | | | | | 8.4 |
|  | |  |  | |  | | | | | O | | | Yonsi | | | | | | | | | | | | | 8.5 |
|  | |  |  | |  | | | | | O | | | Siloano | | | | | | | | | | | | | 8.6 |
|  | |  |  | |  | | | | | O | | | Autre (préciser) : | | | | | | | | | | | | | 8.7 |
|  | |  | O | | Autre (préciser) | | | | | | | | | | | | | | | | | | | | | 50 |
|  | |  | O | | Pas de réponse | | | | | | | | | | | | | | | | | | | | | 99 |
|  | |  |  | | | | | | | | |  | | | |  | |  | | | |  | | |  | |
| **Q17** | | **Coordonnées GPS du point d’eau** | | | | | | | | | | | | | | | | | | | | | | | | |
|  | |  | | | | | No | | | | |  | | | |  | |  | | | |  | | |  | |
|  | |  | | | | |  | | | | |  | | | |  | |  | | | |  | | |  | |
| **Q18** | | **En référence à la question 16, pourquoi choisissez-vous cette source comme source principale ? (plusieurs réponses)** | | | | | | | | | | | | | | | | | | | | | | | | |
|  | |  | O | | ça ne coûte pas trop cher, je peux me le permettre | | | | | | | | | | | | | | | | | | | | | 1 |
|  | |  | O | | C’est facile d’accès | | | | | | | | | | | | | | | | | | | | | 2 |
|  | |  | O | | C’est bien traité / protégée | | | | | | | | | | | | | | | | | | | | | 3 |
|  | |  | O | | Autre (préciser) | | | | | | | | | | | | | | | | | | | | | 50 |
|  | |  | O | | Pas de réponse | | | | | | | | | | | | | | | | | | | | | 99 |
|  | |  |  | | | | | | | | |  | | | |  | |  | | | |  | | |  | |
| **Q19** | | **En référence à la question 16, quand prenez-vous de l’eau de cette source ?** | | | | | | | | | | | | | | | | | | | | | | | | |
|  | |  | O | | Matin | | | | | | | | | | | | | | | | | | | | | 1 |
|  | |  | O | | Midi | | | | | | | | | | | | | | | | | | | | | 2 |
|  | |  | O | | Soir | | | | | | | | | | | | | | | | | | | | | 3 |
|  | |  | O | | Pas de réponse | | | | | | | | | | | | | | | | | | | | | 99 |
|  | |  |  | | | | | | | | |  | | | |  | |  | | | |  | | |  | |
| **Q20** | | **Quelle est votre source secondaire d’eau (de boisson) ? (une seule réponse)** | | | | | | | | | | | | | | | | | | | | | | | | |
|  | |  | O | | L’eau du robinet dans mon habitation | | | | | | | | | | | | | | | | | | | | | 1 |
|  | |  | O | | L’eau du robinet aux points de distribution communaux (REGIDESO) | | | | | | | | | | | | | | | | | | | | | 2 |
|  | |  | O | | Vendeurs dans la rue | | | | | | | | | | | | | | | | | | | | | 3 |
|  | |  | O | | Source protégée | | | | | | | | | | | | | | | | | | | | | 4 |
|  | |  | O | | Source non protégée | | | | | | | | | | | | | | | | | | | | | 5 |
|  | |  | O | | Puits équipé d’une pompe à main | | | | | | | | | | | | | | | | | | | | | 6 |
|  | |  | O | | Puits non équipé d’une pompe à main | | | | | | | | | | | | | | | | | | | | | 7 |
|  | |  | O | | Eau de surface – Préciser le nom du cours d’eau : | | | | | | | | | | | | | | | | | | | | | 8 |
|  | |  |  | |  | | | | | O | | | Kwilu | | | | | | | | | | | | | 8.1 |
|  | |  |  | |  | | | | | O | | | Lukemi | | | | | | | | | | | | | 8.2 |
|  | |  |  | |  | | | | | O | | | Luini | | | | | | | | | | | | | 8.3 |
|  | |  |  | |  | | | | | O | | | Iba | | | | | | | | | | | | | 8.4 |
|  | |  |  | |  | | | | | O | | | Yonsi | | | | | | | | | | | | | 8.5 |
|  | |  |  | |  | | | | | O | | | Siloano | | | | | | | | | | | | | 8.6 |
|  | |  |  | |  | | | | | O | | | Autre (préciser) : | | | | | | | | | | | | | 8.7 |
|  | |  | O | | Autre (préciser) | | | | | | | | | | | | | | | | | | | | | 50 |
|  | |  | O | | Pas de réponse | | | | | | | | | | | | | | | | | | | | | 99 |
|  | |  | | | | |  | | | | |  | | | |  | |  | | | |  | | |  | |
| **Q21** | | **En référence à la question 20, quand prenez-vous de l’eau de cette source secondaire?** | | | | | | | | | | | | | | | | | | | | | | | | |
|  | |  | O | | Matin | | | | | | | | | | | | | | | | | | | | | 1 |
|  | |  | O | | Midi | | | | | | | | | | | | | | | | | | | | | 2 |
|  | |  | O | | Soir | | | | | | | | | | | | | | | | | | | | | 3 |
|  | |  | O | | Pas de réponse | | | | | | | | | | | | | | | | | | | | | 99 |
|  | |  |  | | | | | | | | |  | | | |  | |  | | | |  | | |  | |
| **Q22** | | **Aujourd’hui, avez-vous traité votre eau de boisson ?** | | | | | | | | | | | | | | | | | | | | | | | | |
| Si « Oui », passez à la question 23  Si « Non », passez directement à la question 24 | | | | | | | O | | Oui | | | | | | | | | | | | | | | | | 1 |
|  |  |  |  |  |  |  | O | | Non | | | | | | | | | | | | | | | | | 2 |
|  |  |  |  |  |  |  | O | | Autre (préciser) | | | | | | | | | | | | | | | | | 50 |
|  |  |  |  |  |  |  | O | | Pas de réponse | | | | | | | | | | | | | | | | | 99 |
|  | |  | | | | |  | | | | |  | | | |  | |  | | | |  | | |  | |
| **Q23** | | **Quel traitement avez-vous appliqué ? (plusieurs reponses)** | | | | | | | | | | | | | | | | | | | | | | | | |
|  | |  | | | | | O | | Chlorer l’eau | | | | | | | | | | | | | | | | | 1 |
|  | |  | | | | | O | | Faire bouillir l’eau | | | | | | | | | | | | | | | | | 2 |
|  | |  | | | | | O | | Filtrer l’eau | | | | | | | | | | | | | | | | | 3 |
|  | |  | | | | | O | | Autre (préciser) | | | | | | | | | | | | | | | | | 50 |
|  | |  | | | | | O | | Pas de réponse | | | | | | | | | | | | | | | | | 99 |
|  | |  | | | | |  | | | | |  | | | |  | |  | | | |  | | |  | |
| **Q24** | | **Quelle source de l’eau utilisez-vous pour les activités suivantes : (une seule reponse)** | | | | | | | | | | | | | | | | | | | | | | | | |
|  |  | | | | | | | | | | | | | | | | Cuisiner | Boire | | | Laver la vaisselle | | | Prendre un bain | | |
|  | L’eau du robinet dans l’habitation | | | | | | | | | | | | | | | | O | O | | | O | | | O | | |
|  | L’eau du robinet des points de distribution | | | | | | | | | | | | | | | | O | O | | | O | | | O | | |
|  | Vendeurs dans la rue | | | | | | | | | | | | | | | | O | O | | | O | | | O | | |
|  | Source protégée | | | | | | | | | | | | | | | | O | O | | | O | | | O | | |
|  | Source non protégée | | | | | | | | | | | | | | | | O | O | | | O | | | O | | |
|  | Puits équipé d’une pompe à main | | | | | | | | | | | | | | | | O | O | | | O | | | O | | |
|  | Puits non équipé d’une pompe à main | | | | | | | | | | | | | | | | O | O | | | O | | | O | | |
|  | Eau de surface | | | | | | | | | | | | | | | | O | O | | | O | | | O | | |
|  | Eau de pluie | | | | | | | | | | | | | | | | O | O | | | O | | | O | | |
|  | |  | | | | |  | | | | |  | | | | |  |  | | |  | | |  | | |
| **Q25** | | **Votre source d’eau de boisson fournit-elle de l’eau tout au long de l’année ?** | | | | | | | | | | | | | | | | | | | | | | | | |
|  | |  | | | | | O | | Oui | | | | | | | | | | | | | | | | | 1 |
|  | |  | | | | | O | | Non | | | | | | | | | | | | | | | | | 2 |
|  | |  | | | | | O | | Autre (préciser) | | | | | | | | | | | | | | | | | 50 |
|  | |  | | | | | O | | Pas de réponse | | | | | | | | | | | | | | | | | 99 |
|  | |  | | | | |  | | | | |  | | | |  | |  | | | |  | | |  | |
| **Q26** | | **Quel récipient utilisez-vous pour conserver TOUTE l’eau de votre ménage ?** | | | | | | | | | | | | | | | | | | | | | | | | |
|  | | *(Plusieurs réponses possibles. « Toute l’eau » signifie l’eau pour tous les usages, y compris pour cuisiner, se laver, etc.)* | | | | | | | | | |  | | | |  | |  | | | |  | | |  | |
|  | |  |  |  |  |  |  |  |  |  |  |  | | |  | | | | | | | Nombre / jour | | | Volume (litres) | |
|  |  |  |  |  |  |  |  |  |  |  |  |  |  |  |  |  |  |  |  |  |  |  |  |  |  |  |
|  | |  | | | | |  | | | | |  | | | Récipients en BIDON | | | | | | |  | | |  | |
|  | |  | | | | |  | | | | |  | | | Option 2 | | | | | | |  | | |  | |
|  | |  | | | | |  | | | | |  | | | Option 3 | | | | | | |  | | |  | |
|  | |  | | | | |  | | | | |  | | | Option 4 | | | | | | |  | | |  | |
|  | |  | | | | |  | | | | |  | | | Option 5 | | | | | | |  | | |  | |
|  | |  | | | | |  | | | | |  | | | Autre (préciser) | | | | | | |  | | |  | |
|  | |  | | | | |  | | | | |  | | | Pas de réponse | | | | | | |  | | |  | |
|  | |  | | | | |  | | | | |  | | | |  | |  | | | |  | | |  | |
| **Q27** | | **A quelle fréquence buvez-vous de l’eau en dehors de chez vous ?** | | | | | | | | | | | | | | | | | | | | | | | | |
| Si « Souvent » ou « Parfois », passer aux questions 29 et 30  Si « Jamais », passer directement à la question 31 | | | | | | | O | | Souvent | | | | | | | | | | | | | | | | | 1 |
|  |  |  |  |  |  |  | O | | Parfois | | | | | | | | | | | | | | | | | 2 |
|  |  |  |  |  |  |  | O | | Jamais | | | | | | | | | | | | | | | | | 3 |
|  |  |  |  |  |  |  | O | | Autre (préciser) | | | | | | | | | | | | | | | | | 50 |
|  |  |  |  |  |  |  | O | | Pas de réponse | | | | | | | | | | | | | | | | | 99 |
|  | |  | | | | |  | | | | |  | | | |  | |  | | | |  | | |  | |
| **Q28** | | **Quelle est votre source d’eau de boisson principale lorsque vous buvez en dehors de chez vous ? (une seule reponse)** | | | | | | | | | | | | | | | | | | | | | | | | |
|  | |  | | O | | L’eau du robinet de mon habitation (pas possible) | | | | | | | | | | | | | | | | | | | | 1 |
|  | |  | | O | | L’eau du robinet aux points de distribution communaux (REGIDESO) | | | | | | | | | | | | | | | | | | | | 2 |
|  | |  | | O | | Vendeurs dans la rue | | | | | | | | | | | | | | | | | | | | 3 |
|  | |  | | O | | Source protégée | | | | | | | | | | | | | | | | | | | | 4 |
|  | |  | | O | | Source non protégée | | | | | | | | | | | | | | | | | | | | 5 |
|  | |  | | O | | Puits équipé d’une pompe à main | | | | | | | | | | | | | | | | | | | | 6 |
|  | |  | | O | | Puits non équipé d’une pompe à main | | | | | | | | | | | | | | | | | | | | 7 |
|  | |  | | O | | Eau de surface – Préciser le nom du cours d’eau | | | | | | | | | | | | | | | | | | | | 8 |
|  | |  | |  | |  | | | | | O | | | Kwilu | | | | | | | | | | | | 8.1 |
|  | |  | |  | |  | | | | | O | | | Lukemi | | | | | | | | | | | | 8.2 |
|  | |  | |  | |  | | | | | O | | | Luini | | | | | | | | | | | | 8.3 |
|  | |  | |  | |  | | | | | O | | | Iba | | | | | | | | | | | | 8.4 |
|  | |  | |  | |  | | | | | O | | | Yonsi | | | | | | | | | | | | 8.5 |
|  | |  | |  | |  | | | | | O | | | Siloano | | | | | | | | | | | | 8.6 |
|  | |  | |  | |  | | | | | O | | | Autre (préciser) : | | | | | | | | | | | | 8.7 |
|  | |  | | O | | Autre (préciser) | | | | | | | | | | | | | | | | | | | | 50 |
|  | |  | | O | | Pas de réponse | | | | | | | | | | | | | | | | | | | | 99 |
|  | |  | | | | |  | | | | |  | | | |  | |  | | | |  | | |  | |
| **DEMANDER À VOIR LE PRINCIPAL RÉCIPIENT UTILISÉ POUR CONSERVER L’EAU** | | | | | | | | | | | | | | | | | | | | | | | | | | |
|  | |  | | | | |  | | | | |  | | | |  | |  | | | |  | | |  | |
| **Q29** | | **Observer : l’orifice du récipient pour conserver l’eau de boisson est-il étroit ?** | | | | | | | | | | | | | | | | | | | | | | | | |
|  | |  | | | | | O | | Oui | | | | | | | | | | | | | | | | | 1 |
|  | |  | | | | | O | | Non | | | | | | | | | | | | | | | | | 2 |
|  | |  | | | | | O | | Autre (préciser) | | | | | | | | | | | | | | | | | 50 |
|  | |  | | | | | O | | Pas de réponse | | | | | | | | | | | | | | | | | 99 |
|  | |  | | | | |  | | | | |  | | | |  | |  | | | |  | | |  | |
| **Q30** | | **Observer : le récipient pour conserver l’eau de boisson est-il recouvert ?** | | | | | | | | | | | | | | | | | | | | | | | | |
|  | |  | | | | | O | | Oui | | | | | | | | | | | | | | | | | 1 |
|  | |  | | | | | O | | Non | | | | | | | | | | | | | | | | | 2 |
|  | |  | | | | | O | | Autre (préciser) | | | | | | | | | | | | | | | | | 50 |
|  | |  | | | | | O | | Pas de réponse | | | | | | | | | | | | | | | | | 99 |
|  | |  | | | | |  | | | | |  | | | |  | |  | | | |  | | |  | |
| **SYSTÈMES SANITAIRES** | | | | | | | | | | | | | | | | | | | | | | | | | | |
|  | |  | | | | |  | | | | |  | | | |  | |  | | | |  | | |  | |
| **Q31** | | **Lorsque vous devez déféquer, où allez-vous ? (une seule reponse)** | | | | | | | | | | | | | | | | | | | | | | | | |
| Si « Latrines », passez à la question 32-34 ; autrement, passez directement à la question 37 | | | | | | | O | | Latrines | | | | | | | | | | | | | | | | | 1 |
|  |  |  |  |  |  |  | O | | Cours d’eau | | | | | | | | | | | | | | | | | 2 |
|  |  |  |  |  |  |  | O | | Terrain à l’air libre | | | | | | | | | | | | | | | | | 3 |
|  |  |  |  |  |  |  | O | | Autre (préciser) | | | | | | | | | | | | | | | | | 50 |
|  |  |  |  |  |  |  | O | | Pas de réponse | | | | | | | | | | | | | | | | | 99 |
|  | |  | | | | |  | | | | |  | | | |  | |  | | | |  | | |  | |
| **Q32** | | **Avec combien de ménages partagez-vous vos latrines ?** | | | | | | | | | | | | | | | | | | | | | | | | |
|  | |  | | | | | O | | 1 | | | | | | | | | | | | | | | | | 1 |
|  | |  | | | | | O | | 2-4 | | | | | | | | | | | | | | | | | 2 |
|  | |  | | | | | O | | 5-7 | | | | | | | | | | | | | | | | | 3 |
|  | |  | | | | | O | | > 7 | | | | | | | | | | | | | | | | | 4 |
|  | |  | | | | | O | | Autre (préciser) | | | | | | | | | | | | | | | | | 50 |
|  | |  | | | | | O | | Pas de réponse | | | | | | | | | | | | | | | | | 99 |
|  | |  | | | | |  | |  | | | | | | |  | |  | | | |  | | |  | |
| **Q33** | | **Comment sont conçues les latrines ? (une seule reponse)** | | | | | | | | | | | | | | | | | | | | | | | | |
|  | |  | | | | | O | | Simple fosse | | | | | | | | | | | | | | | | | 1 |
|  | |  | | | | | O | | VIP (latrines améliorées à fosse ventilée) | | | | | | | | | | | | | | | | | 2 |
|  | |  | | | | | O | | Latrines à chasse d’eau | | | | | | | | | | | | | | | | | 3 |
|  | |  | | | | | O | | Latrines à chasse d’eau + fosse septique | | | | | | | | | | | | | | | | | 4 |
|  | |  | | | | | O | | Autre (préciser) | | | | | | | | | | | | | | | | | 50 |
|  | |  | | | | | O | | Pas de réponse | | | | | | | | | | | | | | | | | 99 |
|  | |  | | | | |  | |  | | | | | | |  | |  | | | |  | | |  | |
| **Q34** | | **A quelle fréquence les latrines sont-elle pleines ?** | | | | | | | | | | | | | | | | | | | | | | | | |
|  | |  | | | | | O | | Jamais | | | | | | | | | | | | | | | | | 1 |
|  | |  | | | | | O | | Parfois (spécialement pendant la saison des pluies) | | | | | | | | | | | | | | | | | 2 |
|  | |  | | | | | O | | Très souvent (plusieurs fois par semaine) | | | | | | | | | | | | | | | | | 3 |
|  | |  | | | | | O | | Autre (préciser) | | | | | | | | | | | | | | | | | 50 |
|  | |  | | | | | O | | Pas de réponse | | | | | | | | | | | | | | | | | 99 |
|  | |  | | | | |  | |  | | | | | | |  | |  | | | |  | | |  | |
| **DEMANDER À VOIR LES LATRINES** | | | | | | | | | | | | | | | | | | | | | | | | | | |
| **Q35** | | **Observer : Y a-t-il de l’urine ou des matières fécales sur le sol, les murs, la lunette, etc. des latrines ?** | | | | | | | | | | | | | | | | | | | | | | | | |
|  | |  | | | | | O | | Oui | | | | | | |  | |  | | | |  | | | | 1 |
|  | |  | | | | | O | | Non | | | | | | |  | |  | | | |  | | | | 2 |
| **Q36** | | **Observer : Dispose-t-on d’eau pour se laver les mains à moins de trois mètres des latrines ?** | | | | | | | | | | | | | | | | | | | | | | | | |
|  | |  | | | | | O | | Oui | | | | | | |  | |  | | | |  | | | | 1 |
|  | |  | | | | | O | | Non | | | | | | |  | |  | | | |  | | | | 2 |
|  | |  | | | | |  | |  | | | | | | |  | |  | | | |  | | |  | |
| **Q37** | | **Observer : Quel matériel est disponible aux latrines pour améliorer le lavage des mains?** | | | | | | | | | | | | | | | | | | | | | | | | |
|  | |  | | | | | O | | Savon | | | | | | | | | | | | | | | | | 1 |
|  | |  | | | | | O | | Cendres | | | | | | | | | | | | | | | | | 2 |
|  | |  | | | | | O | | Boue | | | | | | | | | | | | | | | | | 3 |
|  | |  | | | | | O | | Rien | | | | | | | | | | | | | | | | | 4 |
|  | |  | | | | | O | | Autre (préciser) | | | | | | | | | | | | | | | | | 50 |
|  | |  | | | | |  | |  | | | | | | |  | |  | | | |  | | |  | |
| **HYGIÈNE** | | | | | | | | | | | | | | | | | | | | | | | | | | |
|  | | | | | | | | | | | | | | | | | | | | | | | | | | |
| **Q38** | | **Aujourd’hui, y a-t-il du savon dans votre ménage ?** | | | | | | | | | | | | | | | | | | | | | | | | |
| Si « Oui », passer à la question 39  Si « Non », passer directement à la question 40 | | | | | | | O | | Oui | | | | | | |  | |  | | | |  | | | | 1 |
|  |  |  |  |  |  |  | O | | Non | | | | | | |  | |  | | | |  | | | | 2 |
|  |  |  |  |  |  |  | O | | Autre (préciser) | | | | | | |  | |  | | | |  | | | | 50 |
|  |  |  |  |  |  |  | O | | Pas de réponse | | | | | | |  | |  | | | |  | | | | 99 |
|  | |  | | | | |  | |  | | | | | | |  | |  | | | |  | | |  | |
| **Q39** | | **Voulez-vous bien m’apporter votre savon ? (observer)** | | | | | | | | | | | | | | | | | | | | | | | | |
|  | |  | | | | | O | | N’en présente pas | | | | | | | | | | | | | | | | | 1 |
|  | |  | | | | | O | | Présente du savon de lessive | | | | | | | | | | | | | | | | | 2 |
|  | |  | | | | | O | | Le savon est encore dans son emballage (nouveau) | | | | | | | | | | | | | | | | | 3 |
|  | |  | | | | | O | | Présente du savon qui semble avoir été utilisé | | | | | | | | | | | | | | | | | 50 |
|  | |  | | | | | O | | Autre (préciser) | | | | | | | | | | | | | | | | | 99 |
|  | |  | | | | |  | |  | | | | | | |  | |  | | | |  | | |  | |
| **Q40** | | **Vous lavez-vous les mains après avoir déféqué ?** | | | | | | | | | | | | | | | | | | | | | | | | |
| **Si « jamais » passer** | | | | | | | O | | Jamais | | | | | | | | | | | | | | | | | 1 |
| **à le question 42** | | | | | | | O | | Parfois | | | | | | | | | | | | | | | | | 2 |
|  | |  | | | | | O | | Toujours | | | | | | | | | | | | | | | | | 3 |
|  | |  | | | | | O | | Autre (préciser) | | | | | | | | | | | | | | | | | 50 |
|  | |  | | | | | O | | Pas de réponse | | | | | | | | | | | | | | | | | 99 |
|  | |  | | | | |  | |  | | | | | | | | | | | | | | | | |  |
| **Q41 En référence à la question 40, Vous lavez-vous les mains avec du savon ?** | | | | | | | | | | | | | | | | | | | | | | | | | | |
|  | |  | | | | | O | | Jamais | | | | | | |  | |  | | | |  | | | | 1 |
|  | |  | | | | | O | | Parfois | | | | | | |  | |  | | | |  | | | | 2 |
|  | |  | | | | | O | | Toujours | | | | | | |  | |  | | | |  | | | | 3 |
|  | |  | | | | | O | | Autre (préciser) | | | | | | |  | |  | | | |  | | | | 50 |
|  | |  | | | | |  | |  | | | | | | |  | |  | | | |  | | | |  |
| **Q42** | | **(Si chef de ménage est femme) Vous lavez-vous les mains avant de préparer la nourriture ?** | | | | | | | | | | | | | | | | | | | | | | | | |
| **Si « jamais » passer** | | | | | | | O | | Jamais | | | | | | | | | | | | | | | | | 1 |
| **à le question 44** | | | | | | | O | | Parfois | | | | | | | | | | | | | | | | | 2 |
|  | |  | | | | | O | | Toujours | | | | | | | | | | | | | | | | | 3 |
|  | |  | | | | | O | | Autre (préciser) | | | | | | | | | | | | | | | | | 50 |
|  | |  | | | | | O | | Pas de réponse | | | | | | | | | | | | | | | | | 99 |
|  | |  | | | | |  | |  | | | | | | |  | |  | | | |  | | |  | |
| **Q43   En référence à la question 42, Vous lavez-vous les mains avec du savon ?** | | | | | | | | | | | | | | | | | | | | | | | | | | |
|  | |  | | | | | O | | Jamais | | | | | | |  | |  | | | |  | | | | 1 |
|  | |  | | | | | O | | Parfois | | | | | | |  | |  | | | |  | | | | 2 |
|  | |  | | | | | O | | Toujours | | | | | | |  | |  | | | |  | | | | 3 |
|  | |  | | | | | O | | Autre (préciser) | | | | | | |  | |  | | | |  | | | | 50 |
|  | |  | | | | | O | | Pas de réponse | | | | | | |  | |  | | | |  | | | | 99 |
| **Q44** | | **(Si la mère s’occupe d’un enfant) Vous lavez-vous les mains après avoir pris soin de votre enfant ?** | | | | | | | | | | | | | | | | | | | | | | | | |
| **Si « jamais » passer** | | | | | | | O | | Jamais | | | | | | | | | | | | | | | | | 1 |
| **à le question 46** | | | | | | | O | | Parfois | | | | | | | | | | | | | | | | | 2 |
|  | |  | | | | | O | | Toujours | | | | | | | | | | | | | | | | | 3 |
|  | |  | | | | | O | | Autre (préciser) | | | | | | | | | | | | | | | | | 50 |
|  | |  | | | | | O | | Pas de réponse | | | | | | | | | | | | | | | | | 99 |
|  | |  | | | | |  | |  | | | | | | |  | |  | | | |  | | | |  |
| **Q45** | | **En référence à la question 44, Vous lavez-vous les mains avec du savon ?** | | | | | | | | | | | | | | | | | | | | | | | | |
|  | |  | | | | | O | | Jamais | | | | | | |  | |  | | | |  | | | | 1 |
|  | |  | | | | | O | | Parfois | | | | | | |  | |  | | | |  | | | | 2 |
|  | |  | | | | | O | | Toujours | | | | | | |  | |  | | | |  | | | | 3 |
|  | |  | | | | | O | | Autre (préciser) | | | | | | |  | |  | | | |  | | | | 50 |
|  | |  | | | | | O | | Pas de réponse | | | | | | |  | |  | | | |  | | | | 99 |
|  | |  | | | | |  | |  | | | | | | |  | |  | | | |  | | | |  |
| **HYGIÈNE RELATIVE À LA NOURRITURE** | | | | | | | | | | | | | | | | | | | | | | | | | | |
|  | |  | | | | |  | |  | | | | | | |  | |  | | | |  | | |  | |
| **Q46** | | **Combien de fois par semaine visitez-vous le marché ?** | | | | | | | | | | | | | | | | | | | | | | | | |
|  | | Numéro hebdomadaire (entrez 99 si inconnu) | | | | | | | | | | | | | | | | | | _________________ | | | | | |  |
|  | |  | | | | |  | | | | | | | | | | | | |  | | | | | |  |
| **Q47** | | **Consommez-vous souvent des aliments provenant d’un marché de plein air ?** | | | | | | | | | | | | | | | | | | | | | | | | |
|  | |  | | | | | O | | Oui | | | | | | | | | | | | | | | | | 1 |
|  | |  | | | | | O | | Non | | | | | | | | | | | | | | | | | 2 |
|  | |  | | | | | O | | Autre (préciser) | | | | | | | | | | | | | | | | | 50 |
|  | |  | | | | | O | | Pas de réponse | | | | | | | | | | | | | | | | | 99 |
|  | |  | | | | |  | |  | | | | | | |  | |  | | | |  | | |  | |
| **Q48** | | **Consommez-vous les aliments froids ou non chauffés? (une seule réponse)** | | | | | | | | | | | | | | | | | | | | | | | | |
|  | |  | | | | | O | | Non, je mange toujours la nourriture chaude ou réchauffée | | | | | | | | | | | | | | | | | 1 |
|  | |  | | | | | O | | Oui, occasionnellement | | | | | | | | | | | | | | | | | 2 |
|  | |  | | | | | O | | Oui, régulièrement | | | | | | | | | | | | | | | | | 3 |
|  | |  | | | | | O | | Autre (préciser) | | | | | | | | | | | | | | | | | 50 |
|  | |  | | | | | O | | Pas de réponse | | | | | | | | | | | | | | | | | 99 |
|  | |  | | | | |  | |  | | | | | | |  | |  | | | |  | | |  | |
| **Q49** | | **Consommez-vous des légumes crus ?** | | | | | | | | | | | | | | | | | | | | | | | | |
|  | |  | | | | | O | | Jamais | | | | | | | | | | | | | | | | | 1 |
|  | |  | | | | | O | | Oui, occasionnellement | | | | | | | | | | | | | | | | | 2 |
|  | |  | | | | | O | | Oui, régulièrement | | | | | | | | | | | | | | | | | 3 |
|  | |  | | | | | O | | Autre (préciser) | | | | | | | | | | | | | | | | | 50 |
|  | |  | | | | | O | | Pas de réponse | | | | | | | | | | | | | | | | | 99 |
|  | |  | | | | |  | |  | | | | | | |  | |  | | | |  | | |  | |
| **Q50** | | **Au cours des repas, partagez-vous l’assiette avec les convives ?** | | | | | | | | | | | | | | | | | | | | | | | | |
|  | |  | | | | | O | | Jamais | | | | | | | | | | | | | | | | | 1 |
|  | |  | | | | | O | | Oui, occasionnellement | | | | | | | | | | | | | | | | | 2 |
|  | |  | | | | | O | | Oui, régulièrement | | | | | | | | | | | | | | | | | 3 |
|  | |  | | | | | O | | Autre (préciser) | | | | | | | | | | | | | | | | | 50 |
|  | |  | | | | | O | | Pas de réponse | | | | | | | | | | | | | | | | | 99 |
| **HYGIÈNE GÉNÉRALE** | | | | | | | | | | | | | | | | | | | | | | | | | | |
|  | |  | | | | |  | |  | | | | | | |  | |  | | | |  | | |  | |
| **Q51** | | **Observer : L’hygiène générale dans le ménage** | | | | | | | | | | | | | | | | | | | | | | | | |
|  | |  | | | | | O | | Il fait propre (pas de vaisselle par terre, pas d’accumulation de déchets, les habitants portent des vêtements propres, etc.) | | | | | | | | | | | | | | | | | 1 |
|  | |  | | | | | O | | Il ne fait pas propre | | | | | | | | | | | | | | | | | 2 |
|  | |  | | | | | O | | Autre (préciser) | | | | | | | | | | | | | | | | | 50 |
|  | |  | | | | | O | | Pas de réponse | | | | | | | | | | | | | | | | | 99 |
|  | |  | | | | | | | | | | | | | | | | | | | | | | | | |
| **Q52** | | **Observer : La cuisine est-elle carrelée ?** | | | | | | | | | | | | | | | | | | | | | | | | |
|  | |  | | | | | O | | Oui | | | | | | | | | | | | | | | | | 1 |
|  | |  | | | | | O | | Non | | | | | | | | | | | | | | | | | 2 |
|  | |  | | | | | O | | Cuisine en plein air | | | | | | | | | | | | | | | | | 3 |
|  | |  | | | | | O | | Autre (préciser) | | | | | | | | | | | | | | | | | 50 |
|  | |  | | | | | O | | Pas de réponse | | | | | | | | | | | | | | | | | 99 |
